# A novel COE-D8-fosfomycin conjugate effectively combats first-line antibiotic-resistant uropathogenic *Escherichia coli*

**DOI:** 10.1371/journal.pone.0352997

**Published:** 2026-07-08

**Authors:** Lin Ruan, Cun Fan, Jingjie Chen, Yingxia Wang, Yuji Ren, Wenli Yuan, Xiaozhe Xiong, Chunming Guo

**Affiliations:** 1 School of Life Sciences, Yunnan University(YNU), Kunming, China; 2 Yunnan Key Laboratory of Cell Metabolism and Disease, Yunnan, China; 3 Guangzhou Laboratory, Guangzhou, China; 4 The Department of Pathology, First affiliated Hospital of Kunming Medical University, Kunming, China; 5 The Department of Clinical Laboratory, First affiliated Hospital of Kunming Medical University, Kunming, China; 6 Department of Clinical Laboratory, The Affiliated Hospital of Yunnan University (The Second People’s Hospital of Yunnan Province), Kunming, China; Amity University, INDIA

## Abstract

Recurrent urinary tract infections (UTIs) caused by multidrug-resistant uropathogenic *Escherichia coli* (UPEC) demand novel antimicrobial approaches. This study evaluated the membrane-intercalating conjugated oligoelectrolyte COE-D8 against 93 clinical UPEC isolates. While >75% of isolates exhibited high-level resistance to first-line antibiotics (MIC > 512 μg/mL), 94.6% remained susceptible to COE-D8 at ≤32 μg/mL. COE-D8 eliminated bacteria fourfold faster than standard antibiotics through membrane disruption driven by a dimensional mismatch with the lipid bilayer. To mitigate the inherent cytotoxicity of COE-D8 alone, we developed a synergistic co-administration strategy with fosfomycin. This approach achieved a significant dose-sparing effect, rescuing 7-day murine survival from 40% to 80% in safety models without compromising efficacy. Furthermore, resistance induction studies identified a high barrier to adaptation, with survival primarily mediated by mzrA mutations. These findings establish the COE-D8/fosfomycin combination as a host-friendly, mechanism-driven strategy against refractory UPEC.

## Introduction

Urinary Tract Infections (UTIs) are among the most prevalent bacterial infections worldwide and significantly impact patient quality of life, particularly in women [1]. As the second most common infectious disease across all human populations [2,3], UTIs frequently become recurrent, with 30% to 50% of patients experiencing reinfection even after standard antibiotic treatment [4]. Uropathogenic *Escherichia coli* (UPEC) is the primary pathogen responsible for these persistent infections. UPEC invades bladder epithelial cells (BECs) via specialized spindle vesicles [5]. By escaping these vesicles and replicating within the host cytoplasm, UPEC forms intracellular bacterial communities (IBCs) containing over 10,000 bacteria—a strategy that facilitates immune evasion and physical protection against antibiotic penetration, directly driving recurrent infection [6–12].

Fosfomycin represents a globally approved first-line clinical agent for these infections [13–16]. Because of its unique chemical structure and minimal cross-resistance with other antibiotic classes, it has gained increasing interest for treating multidrug-resistant (MDR) UTIs [17–21]. Besides fosfomycin, other guideline-recommended first-line UTI agents include nitrofurantoin, cephalothin, and sulfamethoxazole, while ampicillin, ofloxacin, and kanamycin are also widely used as empirical or secondary therapeutic options. Despite the use of these antibiotics, resistance among UPEC strains has risen continuously and has become a major global clinical challenge. This resistance now extends to nearly all classes of antibiotics, including recently developed β-lactams like oxacillin, flucloxacillin, and dicloxacillin [22].

Worryingly, no new class of antibiotics has been approved for the treatment of Gram-negative bacterial (GNB) infections in over 50 years [23]. Antimicrobial peptides (AMPs) can kill bacteria primarily via membrane or cell wall destabilization, a mode of action that mitigates the risk of resistance development [24–26]. However, their inherent host cytotoxicity severely limits their clinical translation [27,28]. To address these dual barriers of antibiotic resistance and host toxicity, co-administration strategies combining novel membrane-active antimicrobials with clinically approved antibiotics have emerged as a promising research direction.

Conjugated oligoelectrolytes (COEs) are a novel class of membrane-active antimicrobial agents with potent antibacterial activity; for example, select COEs exhibit superior *in vitro* efficacy against methicillin-resistant *Staphylococcus aureus* (MRSA) compared to vancomycin [29]. Structurally, COEs consist of a hydrophobic, linear conjugated backbone with terminal ionic groups, an architecture that closely matches the distribution of charged and hydrophobic domains in lipid bilayers. This structure allows COEs to spontaneously intercalate into cellular membranes [30]. The molecular length of a COE directly determines its functional effects on lipid bilayers after intercalation [31]. As shown in S1 Fig, COE-D8 is a representative and potent antimicrobial molecule, whose activity arises from membrane disruption driven by a dimensional mismatch between the COE-D8 molecular length and the lipid bilayer thickness [32]. Based on this membrane-targeting mechanism, we hypothesized that COE-D8 exerts bactericidal activity against clinical MDR-UPEC via direct cell membrane disruption.

The objectives of this study were to systematically characterize the *in vitro* antibacterial activity, bactericidal mechanism, and adaptive resistance profiles of COE-D8 against clinical MDR-UPEC isolates. We also evaluated the preclinical safety of a COE-D8 and fosfomycin co-administration strategy in non-infectious animal models to lay a foundational framework for subsequent *in vivo* evaluation. Here, we report a synergistic interaction between the membrane-disrupting agent COE-D8 and the cell wall synthesis inhibitor fosfomycin. This combination reduces the host cytotoxicity of COE-D8 while enhancing its antimicrobial efficacy against MDR-UPEC, providing a promising preclinical strategy to address recurrent, multidrug-resistant UPEC infections.

## Materials and methods

### Materials, strains and instruments

The COE-D8 used in this study was synthesized according to previous literature protocols [29,33]. The following drugs were purchased from MCE: colistin, polymyxin B, ampicillin, kanamycin, fosfomycin, cephalothin, sulfamethoxazole, levofloxacin, and nitrofurantoin. Experimental mice were obtained from the Animal Center of Yunnan University, Kunming, China (Ethics approval number: CHSRE20220307). Ninety-three strains of multidrug-resistant Uropathogenic *Escherichia coli* (MDR-UPEC) were isolated from urine culture plates of patients in the Department of Urology, The First Affiliated Hospital of Kunming Medical University. Mueller-Hinton-Broth (MHB, Solarbio), Lysogeny Broth (LB, Solarbio), and 1 × Phosphate-Buffered Saline (PBS, pH 7.2) were prepared and autoclaved before use. The 3T3 cell line, purchased from Pricella (Wuhan, China), was used as a mammalian cell model in the *in vitro* cytotoxicity assays. Hematoxylin and eosin (H&E)-stained tissue sections were prepared using a paraffin embedding machine (MEDITE TES99), followed by visualization with a Slide Scanning Imaging System (SQS40P/SQS40PRO; Shenzhen Shengqiang Technology Co., Ltd.).

### Bacterial isolation, culture and preservation

Primary MDR-UPEC isolates (n = 93) collected from clinical culture plates were divided into two aliquots. Half of the primary cells were directly cryopreserved in 50% glycerol at −80 °C (designated as the “O” series). The remaining cell were expanded in LB at 37 °C for 16 h and subsequently cryopreserved as the working “L” series. For all downstream analyses, “L” series stocks were revived in LB or MHB media. Cultures were subcultured to the mid-logarithmic phase, serially diluted, and plated on LB agar. Three independent single colonies per strain were randomly selected for subsequent experiments.

## Method

### Minimum Inhibitory Concentration (MIC) determination

MIC values were determined using a broth microdilution method. Bacterial cells were cultured in MHB at 37 °C to the mid-log phase (OD_600_ 0.4-0.5) and diluted to a final inoculum of 10^5 CFU/mL. COE-D8 and standard antibiotics (S1 Table) were prepared as 10 mg/mL stock solutions in sterile solvents according to Clinical and Laboratory Standards Institute (CLSI) guidelines. Bacterial suspensions (50 μL) were aliquoted into 96-well microtiter plates and mixed with equal volumes of twofold serially diluted antimicrobial agents. Following 16–18 h of incubation at 37 °C (200 rpm), growth inhibition was assessed by measuring the optical density at 600 nm using a TECAN Infinite M200 microplate reader. The MIC was defined as the lowest concentration of the tested compound that completely inhibited visible bacterial growth.

### Mammalian cell cytotoxicity assay

Biocompatibility was evaluated using the MTT cell viability assay in 3T3 cells [34]. 3T3 cells were seeded into 96-well plate at an initial cell density of 1 × 10^5 cells per well and incubated with COE-D8 (1–512 μg/mL) at 37 °C for 24 h. Following exposure, the medium was discarded, and cells were washed with 1 × PBS. MTT solution (1 mg/mL) was added to each well, and the plates were incubated for an additional 4 h. The supernatant was then carefully removed, and the resulting formazan crystals were dissolved in 100 μL of DMSO per well with orbital shaking (100 rpm, 15 min). Absorbance was measured at 570 nm using a TECAN Infinite M200 microplate reader.

### Time-Kill assay

Bacterial cells were prepared and aliquoted into 96-well plates as described for the MIC assay. The suspensions were challenged with equal volumes (50 μL) of media containing either 4 × MIC COE-D8 or the tested antibiotics for strains 63, 73, 76 and 80, or a combination of 1 × MIC COE-D8 and fosfomycin for strains 73 and 76. Plates were covered with a breathable sealing film and incubated at 37 ℃ with orbital shaking at 200 rpm under aerobic conditions. At predetermined intervals (0, 0.5, 1, 2, 3, 4, 5 and 6 h post-inoculation), 20-μl aliquots were sampled, serially diluted in sterile 1 × PBS, and plated onto LB agar plates. Following an 18-h incubation 37 ℃, colonies were counted to determine the CFU/mL at each time point.

### Evolution of resistance to COE-D8

*In vitro* resistance evolution was performed via serial passaging [25]. UPEC isolates (#63, #74, #76 and #80) cultured to the mid-log phase in MHB,were utilized. Initial inocula (1 × 10^7 CFU/mL) were dispensed into 24-well (1 mL/well) containing gradient concentrations of COE-D8 and incubated at 37 ℃. Cultures were passaged every 24 h; specifically, the culture exhibiting visible growth (OD_600_ ≥ 0.2) at the highest COE-D8 concentration was diluted 1:100 into fresh media containing escalating drug concentrations. Passaging was maintained for 33 days (strains #63 and #76), 19 days (strain #74), or 28 days (strain #80). Endpoint isolates were cryopreserved at −80 °C.

### Outer membrane permeability assay

Outer membrane permeability was assessed using the 1-N-phenylnaphthylamine (NPN) uptake assay [35,36]. Overnight cultures of strains #73 and #76 were diluted to an OD_600_ of 0.01 in fresh MHB and grown to the mid-logarithmic phase (OD_600_ ≈ 0.5) at 37 °C (200 rpm). Cells were collected by centrifugation at 3,000 × g for 10 min, washed twice, and resuspended to 10^7 CFU/mL in 5 mM HEPES buffer. Bacterial suspensions (100 μL) were mixed with 40 μM NPN (50 μL) in a black 96-well plate (Corning Costar). Following the addition of 50 µL COE-D8 at specified concentrations, fluorescence was immediately recorded (excitation: 350 nm, emission: 420 nm) using a TECAN Infinite F200 microplate reader. Appropriate vehicle and NPN background controls were included.

### Animal ethics

All animal experiments were performed in strict compliance with the current animal welfare regulations and guidelines, including the Guide for the Care and Use of Laboratory Animals (NIH Publication no. 85−23, revised 1996). The study protocol was reviewed and approved by the Experimental Animal Management and Use Committee (IACUC) of Yunnan University, Kunming, China (Permit No. CHSRE2022030). Mice were obtained from the Animal Center of Yunnan University and housed under controlled environmental conditions (temperature: 22 ± 2 °C; relative humidity: 50 ± 10%; 12-hour light/dark cycle) with *ad libitum* access to standard laboratory chow and water.

### *In vivo* toxicity assay

Following a 7-day acclimation period, mice were randomly allocated to three experimental groups and three control groups, with n = 5 mice per group. Mice were housed at a density of no more than 5 per cage with adequate space. Baseline body weight was recorded prior to intraperitoneal (i.p.) drug administration (0 h), and body weight was measured daily for a 7-day period. Experimental groups included: (1) 25 mg/kg COE-D8 (dissolved in DMSO), (2) 50 mg/kg fosfomycin (dissolved in PBS), (3) COE-D8 (25 mg/kg) + fosfomycin (50 mg/kg), and solvent control groups (DMSO, PBS, and DMSO + PBS). Humane endpoints were predefined to ensure ethical compliance, mandating immediate euthanasia by CO2 inhalation for animals exhibiting ≥20% baseline weight loss, lethargy, inappetence, or other signs of severe distress. Daily welfare assessments were conducted between 9 a.m. and 12 noon. At the study endpoint, blood samples were collected by retro-orbital puncture under isoflurane anesthesia, and all mice were then euthanasia of by CO_2_ inhalation. Whole blood samples were storage at 4 °C for 16 hours, then centrifuged at 3,000 rpm for 15 min at 6 °C to isolate serum. Serum levels of ALT, AST, and BUN were measured using an automated biochemical analyzer (Wuhan Servicebio Technology Co., Ltd.).

### Tissue preparation and paraffin embedding

Mice were euthanized by CO_2_ asphyxiation. The kidneys, liver, and spleen were harvested and fixed in 4% paraformaldehyde (4mL) overnight at 4°C. Following fixation, the tissues were dehydrated through a graded ethanol series (75%, 85%, and 95%) and immersed twice in 100% ethanol for 1 h each. Subsequently, the tissues were cleared in xylene twice for 1 h each. The samples were then infiltrated with paraffin wax (two changes, 1 h each) and embedded into paraffin blocks. The embedded tissues were sectioned at a thickness of 5 μm and mounted onto glass slides.

### Hematoxylin and Eosin (H&E) staining

Paraffin sections were baked at 65 °C for 2 h to ensure tissue adhesion. The sections were deparaffinized in deparaffinizing solution(two changes, 10 min each). Tissues were rehydrated through a graded ethanol series (100% twice, 95%, and 75% for 10 min each). The sections were stained with hematoxylin, followed by treatment with differentiation and bluing reagents, and then rinsed three times under running tap water. Counterstaining was performed with eosin for 1 min, and the sections were subsequently dehydrated in 95% and 100% ethanol. The samples were cleared in a clearing agent for 4 min and mounted using a xylene-based mounting medium.

### Bioinformatics

Genomic DNA was extracted from isolated E.coli strains and subjected to 150-bp paired-end sequencing on an Illumina NovaSeq 6000 platform (Major Biotechnology, Shanghai, China). Raw sequencing data were quality-filtered and adapter-trimmed using fastp (v0.23.2) [37]. High-quality paired-end reads, along with singleton reads that passed quality control, were assembed *de novo* using SPAdes (v3.15.5) [38]. Assembly quality was evaluated using QUAST (v5.2.0) [39], and assemblies with an N50 < 200,000 bp were excluded from downstream analyses.

The remaining assemblies, alongside with 46 reference genomes, were annotated using Prokka (v1.14.6) [40] in fast mode to rapidly identify gene features without assigning functional annotation. The annotated nucleotide sequences, including coding region, tRNAs, and non-coding RNAs, were clustered into orthologous groups using the custom pipeline OrthoSLC (v0.2.4) [41,42]. This orthologous clustering also facilitated the identification of single nucleotide polymorphisms (SNPs) across strains before and after the resistance evolution assays.Multiple sequence alignment of the clustered core genes were performed using Kalign (v3.3.5) [43] with default parameters, and the aligned clusters were concatenated into a core genome sequence for each strain using a custom script. Maximum likelihood phylogenetic trees were constructed using RAxML (v8.0.2) [44] under GTRCAT substitution model with 1,000 rapid bootstraps replicates [45].

For proteins harboring resistance-associated amino acid substitutions, three dimensional structures were predicted using AlphaFold2. Molecular docking of COE-D8 to these predicted protein structures was performed using Autodock Vina (v1.5.7) with “exhaustiveness=20”. Docking conformations were visualized using PyMol(v3.0.5). Data visualization in this study was was conducted using the *ggplot2* (v3.4.3) and *ggtree* (v3.10.0) packages in R. All sequencing data have been deposited in the China National Center For Bioinformation (CNCB) under accession number PRJCA024060.

### COE-D8 synthesis

The synthesis of COE-D8 follows a modular two-step protocol. Briefly, the precursor (E)-1,2-bis(4-(8-iodooctyloxy)phenyl)ethene was first prepared by alkylation of trans-stilbene-4,4’-diol with 1,8-diiodooctane under reflux in acetone using K₂CO₃ as base. Subsequent quaternization with trimethylamine in methanol/THF at 55 °C afforded COE-D8 as an off-white solid in high yield (90%) [32].

## Result

### COE-D8 outperforms conventional antibiotics against multidrug-resistant clinical UPEC isolates

**Fig 1:**
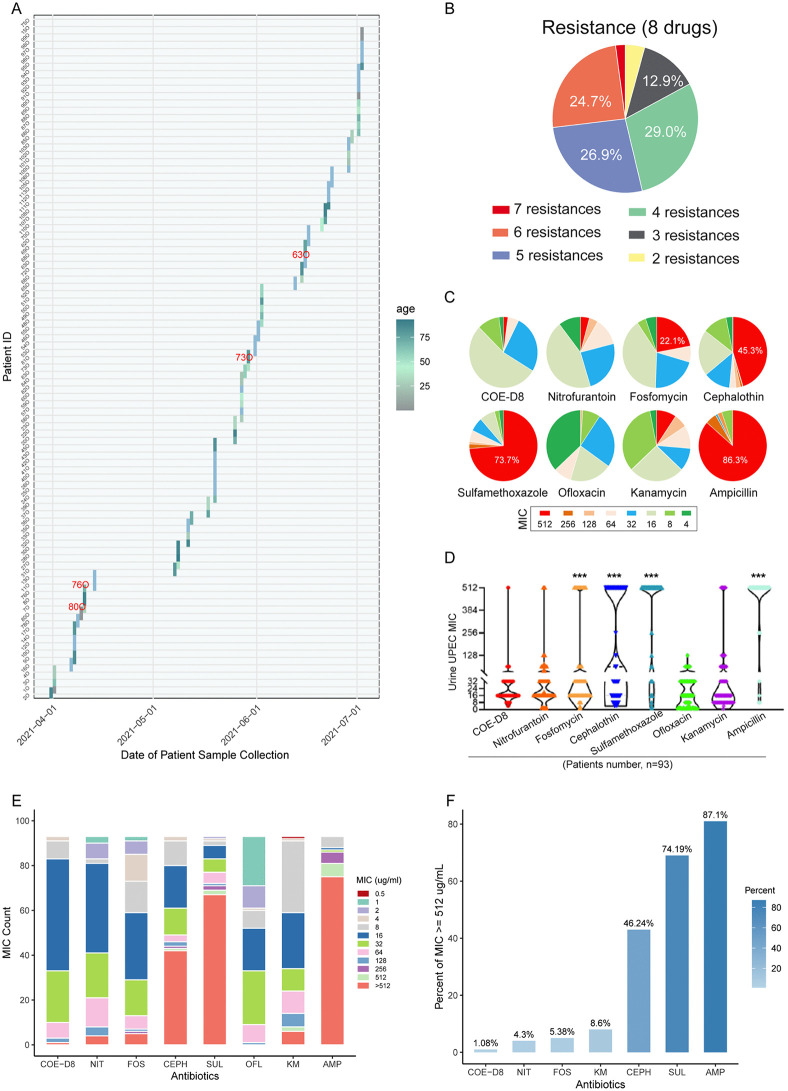
Clinical characteristics and antimicrobial susceptibility profiles of 93 UPEC isolates. (A) Timeline of sample collection from female patients in the urology department of a hospital in Southwest China. Patient IDs (y-axis) are color-coded by age. *(Representative strains labeled as 63O, 73O, 76O, and 80O, selected for subsequent studies, are highlighted in red)*. (B) Pie chart detailing the distribution of resistance frequencies (number of resisted drugs) among the strains. (C) Proportional breakdown of MIC values for UPEC against COE-D8 and seven conventional antibiotics. (D) Violin plots comparing the MIC distributions of COE-D8 with seven conventional antibiotics (nitrofurantoin, fosfomycin, cephalothin, sulfamethoxazole, ofloxacin, kanamycin, and ampicillin). Statistical significance was determined using the Kruskal-Wallis test followed by Dunn’s multiple comparisons test (*** *P* < 0.001 compared to COE-D8). (E) Stacked bar chart detailing the count of MIC values per drug. (F) Proportion of isolates exhibiting an MIC ≥ 512 μg/mL for each tested antibiotic.

A total of 93 Uropathogenic *Escherichia coli* (UPEC) isolates were collected between Mar. 2021 and Aug. 2021. Each isolate was derived from an independent patient, ensuring genetic diversity. Whole-genome sequencing and phylogenetic analysis confirmed the distinct lineages of these isolates (S2 Fig). The isolates were obtained from patients across a broad age range (11 days to 93 years) (Fig 1A). Antimicrobial susceptibility testing was performed for COE-D8 and seven conventional antibiotics used for UTI treatment: nitrofurantoin, fosfomycin, cephalothin, sulfamethoxazole, ofloxacin, kanamycin, and ampicillin (S1 Table). All isolates exhibited resistance to at least two of the antibiotics tested (Fig 1B).

The minimum inhibitory concentrations (MICs) of COE-D8 against the 93 UPEC isolates predominantly clustered at 16 μg/mL, with only one isolate exhibiting an MIC of 512 μg/mL (Fig 1C). In contrast, the MIC distributions for the other seven conventional antibiotics shifted toward higher concentration ranges (Figs 1C and 1D). Specifically, COE-D8 inhibited 94.6% of the isolates (88/93) at concentrations ≤32 μg/mL (Fig 1E). Conversely, high MICs (≥ 512 μg/mL) were frequently observed for sulfamethoxazole (73.7%), cephalothin (45.3%), and fosfomycin (22.1%)(Fig 1F).

### COE-D8 kills phylogenetically diverse UPEC isolates

**Fig 2:**
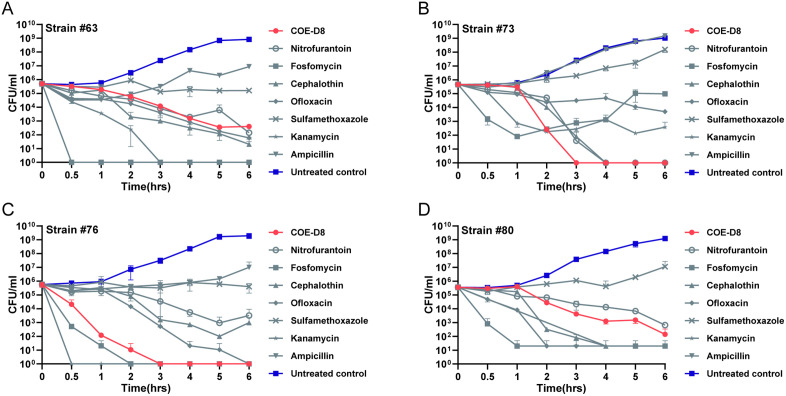
Time-kill assays of COE-D8 and various antibiotics at a concentration of 4 × MIC against: (A) Strain #63; (B) Strain #73; (C) Strain #76; and (D) Strain #80.

While MIC assays establish growth inhibition, they do not determine whether the effect is bactericidal. To assess this, we measured the time-kill kinetics of COE-D8 (at 4 × MIC) against four representative UPEC strains from distinct phylogroups (B2: #73, #76; B1: #63; and E: #80). Upon COE-D8 exposure, viable cell counts for strains #73 and #76 decreased below the detection limit (10^0 CFU/mL)—representing a > 5 log10 reduction—within 3 hours. Over the 6-hour observation period, viable cell counts decreased by >3 log10 for strain #80 and approximately 3 log10 for strain #63. In contrast, the time-kill profiles of the seven conventional antibiotics varied across the isolates. While fosfomycin and kanamycin eliminated detectable viable cells for strains #63 and #76 within 3 hours, treatment with the conventional antibiotics otherwise resulted in static viable counts or minor bacterial regrowth (≤1 log10 increase) over the 6-hour period (Fig 2).

### COE-D8 exerts bactericidal activity by targeting the cell membrane, driving MzrA-mediated adaptive resistance

**Fig 3:**
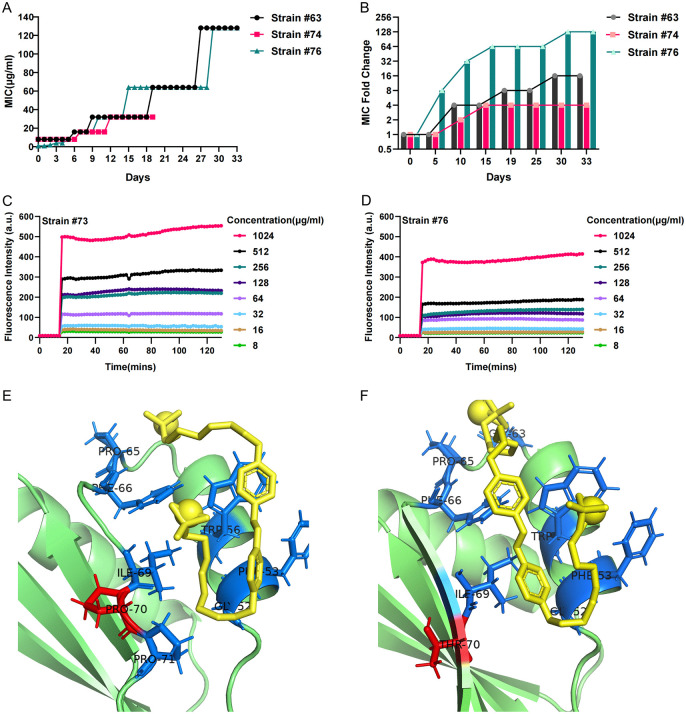
Evolution of COE-D8 resistance and the structural basis of MzrA-mediated adaptation. (A, B) Continuous *in vitro* evolution of COE-D8 resistance in *E. coli* strains #63 (black), #74 (pink), and #76 (teal). (A) Minimum inhibitory concentration (MIC) trajectories over 33 days of serial passaging. (B) Corresponding fold-changes in MIC relative to the original clinical isolates (Day 1). (C, D) Real-time outer membrane permeabilization dynamics assessed by 1-N-phenylnaphthylamine (NPN) uptake. Dose-dependent increases in NPN fluorescence indicate enhanced outer membrane permeability in evolved strains #73 (C) and #76 (D) upon acute COE-D8 exposure (8–1024 μg/mL). (E, F) Structural basis of resistance. Molecular docking of COE-D8 (yellow) to the wild-type (E) and evolved (F) MzrA protein (strain #63 origin). The adaptive P70T substitution is highlighted in red, with key binding pocket residues in blue.

Serial passaging of UPEC strains (#63, #74, #76, and #80) in the presence of sub-lethal, escalating concentrations of COE-D8 resulted in increased MICs. After 33 days of passaging, strain #76 exhibited a 128-fold increase in MIC (from 1 to 128 μg/mL). Strain #63 reached a 16-fold increase over 33 days, while strain #74 exhibited a 4-fold increase by day 19 (Figs 3A and 3B). Strain #80 failed to survive after day 28.

**Fig 4:**
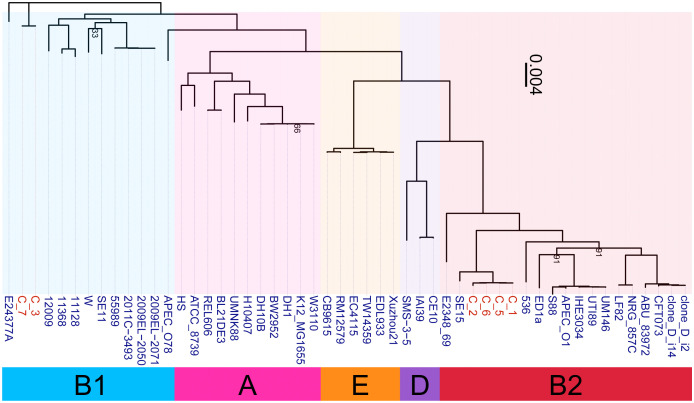
Phylogenetic framework and phylogroup distribution of the evolved COE-D8 resistant *E. coli* strains. High-resolution phylogenetic tree constructed from single-copy core genomes of 46 *E. coli* reference strains (labeled in blue) alongside three paired clinical isolates and their corresponding COE-D8-resistant derivatives (labeled in red). Specifically, C_1, C_2, and C_3 represent the original clinical isolates (#63, #74, and #76, respectively), while C_5, C_6, and C_7 denote their corresponding COE-D8-resistant derivatives. Major clades are background-shaded to delineate the canonical *E. coli* phylogroups (B1, A, E, D, and B2), with corresponding assignments indicated by the bottom color bar. Bootstrap support values < 95% are displayed at the respective internodes. The scale bar represents 0.004 nucleotide substitutions per site.

Whole-genome sequencing of the evolved resistant isolates identified distinct nucleotide variations relative to their wild-type ancestors: the derivative of strain #63 harbored 561 base pair (bp) substitutions across 14 genes, #74 contained 432 bp changes across 15 genes, and #76 exhibited 467 bp changes across 27 genes (S2 Table). Phylogenetic analysis confirmed the isogenic relationships between pre- and post-evolution pairs (Fig 4).To functionally validate the membrane-targeting mechanism, 1-N-phenylnaphthylamine (NPN) uptake assays were performed, revealing a dose-dependent increase in outer membrane permeability upon COE-D8 exposure in strains #73 (Fig 3C) and #76 (Fig 3D).

Structural modeling of the MzrA protein from the evolved isolate (strain #63 origin) revealed that the adaptive Pro70Thr (P70T) substitution altered a local turn into a β-strand (Figs 3E and 3F). Molecular docking simulations reproducibly positioned COE-D8 adjacent to this newly formed β-strand in the mutant MzrA, yielding a binding affinity of −5.0 kcal/mol, compared to −4.6 kcal/mol for the wild-type (S3 Table).

### *In vitro* and *in vivo* safety profile and synergistic bactericidal efficacy of COE-D8 combined with fosfomycin

**Fig 5:**
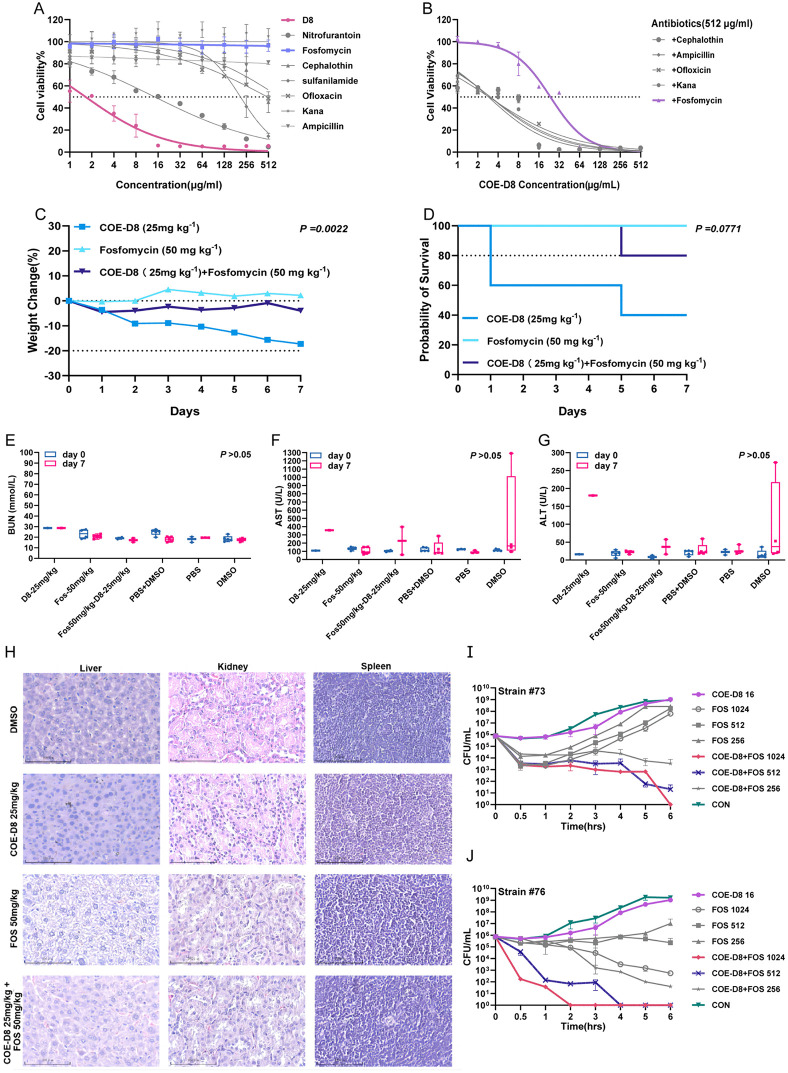
*In vitro* and *in* *vivo* safety profile and synergistic bactericidal efficacy of COE-D8 combined with fosfomycin. (A, B) Mammalian cell cytotoxicity profiles against 3T3 cells. (A) Dose-dependent cell viability under COE-D8 monotherapy compared to conventional antibiotics. (B) Dose-dependent cell viability of COE-D8 combined with a fixed high dose (512 μg/mL) of conventional antibiotics. (C, D) *In vivo* tolerability and survival outcomes. (C) Percentage changes in body weight following intraperitoneal administration of COE-D8 (25 mg/kg), fosfomycin (50 mg/kg), or their combination. Data indicate the mean percentage change (n = 5 mice/group) with standard deviation (SD). The treatment effect was highly significant (Repeated-measures ANOVA with Greenhouse-Geisser correction: *F*(1.12, 7.82) = 19.01, *P* = 0.0022, η² = 0.73; Tukey’s post hoc *P* < 0.05 for all pairwise comparisons). (D) Kaplan-Meier survival curves over the 7-day safety evaluation period following drug administration, revealing no significant differences in mortality across treatment groups (Log-rank Mantel-Cox test: *χ²* = 5.12, df = 2, *P* = 0.077). (E-G) Hepatic and renal toxicity biomarkers. Serum levels of (E) blood urea nitrogen (BUN), (F) aspartate aminotransferase (AST), and (G) alanine aminotransferase (ALT) were quantified on Day 0 and Day 7 across six experimental groups (including drug treatments and vehicle controls: PBS+DMSO, PBS, DMSO). Box plots show individual values, means, and SD. Differences between Day 0 and Day 7 across the six conditions (n = 6 paired groups) were not statistically significant (Two-tailed paired t-tests: BUN *P* = 0.1985, AST *P* = 0.1367, ALT *P* = 0.1233). (H) Histopathological evaluation. Representative H&E-stained sections of the liver, kidney, and spleen harvested at Day 7 post-treatment, showing preserved tissue architecture. Scale bar = 200 μm. (I, J) Synergistic time-kill kinetics. Bactericidal curves of COE-D8 (16 μg/mL) combined with escalating concentrations of fosfomycin (256, 512, and 1024 μg/mL) against (I) clinical strains #73 and (J) #76.

Mammalian cytotoxicity assays in 3T3 cells determined the IC₅₀ of COE-D8 alone to be approximately 6 μg/mL, whereas conventional antibiotics exhibited IC₅₀ values ≥512 μg/mL (Fig 5A). Concurrent exposure of COE-D8 with fosfomycin (512 μg/mL) shifted the IC₅₀ of COE-D8 to >16 μg/mL (Fig 5B).

In the mouse model, administration of COE-D8 alone (25 mg/kg) resulted in a continuous decrease in body weight (approaching −20% by day 7). In contrast, the COE-D8 and fosfomycin combination group maintained a stable body weight comparable to the fosfomycin-only group (Fig 5C). Over the 7-day observation period, the survival rate was 40% for the group administered COE-D8 alone, compared to 80% for the combination group (Fig 5D). Serum biomarker analysis revealed that blood urea nitrogen (BUN) levels remained stable across all groups (Fig 5E). Furthermore, the elevations in hepatic AST and ALT levels induced by single-agent COE-D8 were visually attenuated in the combination group (Figs 5F and 5G). Histopathological evaluation of the liver, kidney, and spleen at day 7 showed preserved tissue architecture across the evaluated groups (Fig 5H).

In time-kill assays against strains #73 and #76, individual exposure to COE-D8 (16 μg/mL) or fosfomycin (256–1024 μg/mL) either resulted in bacterial regrowth or merely maintained bacteriostatic suppression without complete clearance. Conversely, co-incubation of COE-D8 with fosfomycin demonstrated a dose-dependent synergistic bactericidal effect. Specifically, the combination containing the highest fosfomycin concentration (1024 μg/mL) rapidly decreased viable cell counts below the detection limit (10^0 CFU/mL) within 2 hours for strain #76 and 6 hours for strain #73. When the fosfomycin concentration was reduced to 512 μg/mL, this complete clearance was achieved in strain #76 within 4 hours, whereas viable counts for strain #73 remained slightly above the detection limit at the 6-hour endpoint. The lowest combination dose (256 μg/mL fosfomycin) exhibited enhanced bacterial reduction relative to individual exposures but failed to eradicate either strain (Figs 5I and 5J).

## Discussion

The increasing prevalence of multidrug resistance among uropathogenic *Escherichia coli* (UPEC) poses a major clinical management of urinary tract infections (UTIs), driven by the progressive loss of efficacy of conventional first-line antibiotics. For more than 50 years, no new class of antibiotics has been approved for the treatment of Gram-negative bacterial infections associated with UTIs, and the clinical translation of membrane-active antimicrobial agents is largely limited by their inherent cytotoxicity. This gap highlights an urgent unmet need for antimicrobial strategies with optimized safety profiles. In this study, we systematically characterized the *in vitro* antimicrobial activity and preclinical safety of the conjugated oligoelectrolyte COE-D8 using 93 clinical multidrug-resistant UPEC isolates. The minimum inhibitory concentration (MIC) values of COE-D8 predominantly clustered at 16 μg/mL, with 94.6% of these genetically diverse isolates inhibited at concentrations ≤32 μg/mL. By comparison, high-level resistance (MIC ≥ 512 μg/mL) was detected in 22.1% to 86.3% of the same isolate cohort for conventional first-line UTI antibiotics, including fosfomycin, cephalothin, sulfamethoxazole, and ampicillin.

Beyond static growth inhibition, COE-D8 exhibited rapid bactericidal kinetics, achieving bacterial elimination at least fourfold faster than standard antibiotics *in vitro*. As validated by NPN uptake assays, this potent activity is mechanistically driven by a critical dimensional mismatch between the COE-D8 molecular length and the bacterial outer membrane, which facilitates spontaneous intercalation and irreversible outer membrane disruption. Furthermore, COE-D8 exhibited a relatively high barrier to resistance during long-term sequential passaging *in vitro*, with UPEC circumventing membrane stress primarily through a specific MzrA-mediated structural adaptation (Pro70Thr mutation), identified via whole-genome sequencing and molecular docking validation.

Crucially, while the inherent mammalian cytotoxicity of standalone COE-D8 restricts its clinical translation, we discovered that its combination with fosfomycin significantly mitigated this host toxicity both *in vitro* and in non-infectious murine safety evaluation models. This combinatorial regimen not only improved murine survival in acute systemic toxicity assessments, but also maintained a potent synergistic bactericidal effect against UPEC *in vitro*. Collectively, these findings establish that the COE-D8 and fosfomycin combination is a promising, mechanism-driven candidate, laying a critical preclinical foundation for subsequent *in vivo* therapeutic evaluation against recurrent multidrug-resistant UPEC infections.

The synergistic bactericidal efficacy observed in our *in vitro* time-kill assays can be attributed to the complementary mechanisms of action between the two agents. Mechanistically, fosfomycin exerts its antibacterial effect by inhibiting bacterial cell wall synthesis, thereby compromising the structural integrity of the Gram-negative bacterial cell envelope. *In vitro*, this initial envelope damage lowers the biophysical barrier for COE-D8, facilitating its rapid access to and intercalation into the bacterial outer membrane, where it exerts membrane-disrupting activity via the dimensional mismatch between its molecular length and bilayer thickness. This outer membrane permeabilization effect was directly validated in our NPN uptake assays. Consequently, this combinatorial synergy significantly reduces the effective bactericidal concentration of COE-D8 required for UPEC eradication *in vitro*. Given that the mammalian cytotoxicity of COE-D8 is strictly dose-dependent, as demonstrated by our 3T3 cell viability assays, this dose-sparing effect provides a critical mechanistic basis for mitigating the host toxicity of COE-D8.

Beyond static growth inhibition, COE-D8 exhibited rapid bactericidal kinetics *in vitro*, achieving bacterial elimination at least fourfold faster than standard antibiotics. As validated by NPN uptake assays, this bactericidal activity is mechanistically driven by a critical dimensional mismatch between the COE-D8 molecular length and the bacterial outer membrane lipid bilayer, which facilitates spontaneous intercalation and irreversible outer membrane disruption. However, recognizing that standalone membrane-disrupting activity often correlates with host cytotoxicity, we sought to decouple these effects through a combinatorial strategy. We found that co-administration with fosfomycin effectively mitigates this host toxicity while preserving antimicrobial potency. This synergy arises from complementary mechanisms: fosfomycin inhibits bacterial cell wall synthesis, thereby compromising the structural integrity of the Gram-negative bacterial cell envelope. This initial damage lowers the biophysical barrier for COE-D8, allowing it to access and intercalate into the outer membrane more efficiently. This mechanistic synergy facilitates a significant dose-sparing effect; since our 3T3 cell viability assays confirm that COE-D8 cytotoxicity is strictly dose-dependent, reducing its effective bactericidal concentration directly provides a mechanistic basis for improved safety.

This *in vitro* dose-sparing advantage translated into a direct host-protective effect within our non-infectious murine safety models. At a fixed high dose of COE-D8 (25 mg/kg), concurrent dosing of fosfomycin (50 mg/kg) effectively countered COE-D8-induced systemic toxicity, as evidenced by the stabilization of murine body weight and the suppression of hepatic AST/ALT elevation. Notably, this combinatorial approach resulted in a twofold increase in the 7-day survival rate within safety models, confirming a host-protective effect without interfering with the bactericidal activity. Nevertheless, the residual 20% mortality observed in the combination group underscores the need for further medicinal chemistry optimization. Future rational design should focus on fine-tuning the amphiphilicity and dimensional parameters of the COE backbone to further enhance selective targeting of bacterial envelopes over mammalian membranes.

Parallel to these efficacy and safety evaluations, we comprehensively mapped the adaptive resistance landscape of UPEC to COE-D8. In our *in vitro* evolution study, UPEC strain #80 developed a 32-fold increase in MIC by day 23 but exhibited a complete loss of viability in drug-free medium after 28 days. This terminal growth defect likely reflects an extreme fitness cost or obligate drug dependence, whereby resistance-associated genes remain functionally inactive in the absence of COE-D8. While strain #80 reached an evolutionary dead-end, analysis of surviving resistant lineages provided more tractable insights into adaptation.

A specific Pro70Thr missense mutation in the MzrA protein emerged as a key candidate determinant of resistance. It should be acknowledged that a limitation of this study is the lack of gene expression analysis of *mzrA* under COE-D8 exposure. Follow-up experiments are required to determine whether this resistance is conferred exclusively by the structural consequences of the Pro70Thr substitution or involves changes in *mzrA* gene expression. Nevertheless, the convergence of phenotypic resistance, this specific amino acid alteration, and molecular docking simulations provides multi-faceted evidence supporting the role of *mzrA* [46] in UPEC adaptation.

Beyond *mzrA*, genomic analysis identified additional non-synonymous mutations in genes such as *nlpD* [47], malT [48], and emrE [49]. Although these substitutions did not map to predicted COE-D8 binding pockets, their presence suggests broader, system-level adaptive responses. For instance, altered MalT activity could reduce membrane permeability [50] through downstream regulon modulation, while emrE mutations may alter efflux pump kinetics. Furthermore, variations in *ribB* might sustain metabolic homeostasis under antibiotic stress [51]. The absence of a single, reproducible genetic signature across all lineages may be attributed to the inherent limitations of short-read sequencing in resolving complex copy number alterations (CNAs) and the stochastic nature of convergent evolution [52]. Collectively, these findings highlight that UPEC employs diverse strategies—encompassing membrane remodeling, active efflux, and metabolic rewiring—that operate independently of direct drug-target physical interactions.

In conclusion, this study systematically characterizes the potent *in vitro* bactericidal activity of COE-D8, elucidates its membrane-disrupting mechanism, and delineates its adaptive resistance profiles. Crucially, we establish a fosfomycin co-administration strategy that mitigates COE-D8-induced host cytotoxicity in non-infectious murine safety models while retaining full *in vitro* efficacy. This work establishes the COE-D8 and fosfomycin combinatorial approach as a highly potent, mechanism-driven preclinical candidate co-administration strategy. More broadly, our findings provide an essential preclinical framework for addressing the intractable challenge of recurrent, multidrug-resistant UTIs caused by UPEC.

## Supporting information

S1 Fig Structure of COE-D8. (TIF)

S2 Fig Single-copy core genome based phylogeny of 46 reference and 91 clinical *E. coli* isolates. (TIF)

S1 Table Antibacterial activity of COE-D8 and comparative antibiotics against clinical and reference Uropathogenic *E. coli*(UPEC) strains. Minimum inhibitory concentrations (MIC, μg/mL) are presented for 93 clinical isolates and two reference strains(K-12 and UTI89). Abbreviations: NIT: Nitrofurantoin; FOS: Fosfomycin; CEPH: Cephalothin; SUL: Sulfamethoxazole; OFL: Ofloxacin; KM: Kanamycin; AMP: Ampicillin.
(DOCX)

S2 Table Non-synonymous single base substitutions acquired during *in vitro* resistance evolution. All single base substitutions were non-synonymous mutations coding for either an amino acid substitution.The last column shows the gene before resistance evolution (left of right arrow) mutate to the gene on the right hand side experiencing corresponding evolution day.
(DOCX)

S3 Table Docking Affinity in Autodock Vina. The gene of MzrA shows a lower docking energy of −5.0 kcal/mol than docking with the protein before resistance evolution −4.6 kcal/mol. There are no docking energy change before and after resistance evolution among the gene of MalT, EmrE and RibB.
(DOCX)

S4 Table *In vivo* safety evaluation of COE-D8, fosfomycin, and their combination following intraperitoneal injection in mice. Data are expressed as the mean ± standard deviation (SD) (n = 5 per group). Statistical significance between Day 0 and Day 7 was determined using a two-tailed Student’s t-test (P < 0.05). Reference standard ranges for healthy mice: AST (36.31–235.48 U/L), ALT (10.06–96.47 U/L), and BUN (10.81–34.74 mg/dL). Abbreviations: AST, aspartate aminotransferase; ALT, alanine aminotransferase; BUN, blood urea nitrogen. Statistical analysis was performed using Student’s t-test; differences are considered statistically significant with probability *P* < 0.05.
(DOCX)
