# Supplementary material for: A novel COE-D8-fosfomycin conjugate effectively combats first-line antibiotic-resistant uropathogenic Escherichia coli
Source: PLoS One. 2026 Jul 8;21(7):e0352997. doi: 10.1371/journal.pone.0352997 (PMC13345249; doi:10.1371/journal.pone.0352997)
Supplement: S1 Table — Minimum inhibitory concentrations (MIC, μg/mL) are presented for 93 clinical isolates and two reference strains(K-12 and UTI89). Abbreviations: NIT: Nitrofurantoin; FOS: Fosfomycin; CEPH: Cephalothin; SUL: Sulfamethoxazole; OFL: Ofloxacin; KM: Kanamycin; AMP: Ampicillin. (DOCX) [file pone.0352997.s003.docx]

**S1 Table. Antibacterial activity of COE-D8 and comparative antibiotics against clinical and reference Uropathogenic *E. coli*(UPEC) strains.**

| **MIC (μg/mL)​**​ | | | | | | | | | | |
| --- | --- | --- | --- | --- | --- | --- | --- | --- | --- | --- |
| **NO.** | **COE-D8** | **NIT** | **FOS** | | **CEPH** | | **SUL** | **OFL** | **KM** | **AMP** |
| **Antimicrobial mechanisms** | Membrane disruption | Cell wall,DNA,RNA synthesis inhibitor | Cell wall synthesis inhibitor | | Cell wall synthesis inhibitor | | Competitive inhibitor of enzyme dihydropteroate synthetase | DNA gyrase inhibitor | Protein synthesis inhibitor | Cell wall synthesis inhibitor |
| **K12** | **8** | 16 | 16 | | 16 | | >512 | 1 | 4 | 16 |
| **UTI89** | **16** | 16 | 8 | | 16 | | 16 | 1 | >512 | >512 |
| **YN1** | **32** | 16 | 8 | | 32 | | 16 | 2 | 8 | >512 |
| **YN2** | **32** | 64 | 16 | | 32 | | 32 | 2 | 32 | >512 |
| **YN3** | **64** | 64 | >512 | | >512 | | >512 | 16 | 16 | >512 |
| **YN4** | **32** | 128 | >512 | | >512 | | 16 | 32 | 64 | >512 |
| **YN7** | **64** | 128 | 8 | | 16 | | 32 | 2 | 16 | 8 |
| **YN8** | **16** | 64 | 8 | | >512 | | 16 | 32 | 16 | 512 |
| **YN9** | **32** | 64 | 16 | | 16 | | 16 | 2 | 16 | 32 |
| **YN10** | **>512** | 64 | 16 | | 16 | | >512 | 16 | 32 | 512 |
| **YN11** | **16** | 64 | 4 | | >512 | | 512 | 32 | 64 | 512 |
| **YN12** | **16** | 64 | 16 | | 16 | | >512 | 1 | 16 | 512 |
| **YN13** | **32** | 16 | 16 | | 16 | | >512 | 16 | 8 | 256 |
| **YN14** | **16** | 16 | 16 | | 16 | | >512 | 16 | 8 | 512 |
| **YN15** | **16** | 16 | 16 | | 16 | | >512 | 1 | 64 | 512 |
| **YN16** | **32** | 16 | 16 | | 16 | | >512 | 2 | 8 | 8 |
| **YN17** | **16** | 16 | 2 | | 16 | | >512 | 2 | 16 | 8 |
| **YN20** | **16** | 16 | 8 | | >512 | | >512 | 2 | 8 | >512 |
| **YN21** | **32** | 16 | 16 | | >512 | | >512 | 16 | 8 | >512 |
| **YN23** | **8** | 16 | 8 | | 16 | | >512 | 64 | 8 | >512 |
| **YN24** | **32** | 32 | 64 | | 32 | | 16 | 64 | 8 | >512 |
| **YN25** | **16** | 16 | 2 | | 16 | | >512 | 32 | >512 | >512 |
| **YN26** | **32** | 16 | 2 | | 64 | | >512 | 32 | 0.5 | >512 |
| **YN27** | **16** | 16 | >512 | | >512 | | >512 | 32 | 8 | >512 |
| **YN28** | **32** | 16 | 16 | | 32 | | >512 | 32 | 8 | >512 |
| **YN30** | **64** | 16 | 2 | | 16 | | >512 | 32 | 4 | >512 |
| **YN31** | **32** | 16 | 1 | | 16 | | >512 | 32 | 8 | >512 |
| **YN32** | **16** | 64 | 8 | | 256 | | 128 | 64 | 8 | >512 |
| **YN33** | **16** | 16 | 4 | | >512 | | >512 | 32 | 8 | >512 |
| **YN35** | **16** | 16 | 4 | | 32 | | 256 | 32 | 8 | >512 |
| **YN36** | **16** | 1 | 1 | | >512 | | 32 | 32 | 8 | >512 |
| **YN37** | **32** | 2 | 2 | | >512 | | >512 | 32 | 32 | >512 |
| **YN39** | **32** | 1 | 4 | | 512 | | >512 | 32 | 16 | >512 |
| **YN40** | **>64** | 2 | 2 | | >512 | | >512 | 32 | 64 | >512 |
| **YN41** | **16** | 2 | 4 | | >512 | | >512 | 32 | 16 | >512 |
| **YN42** | **32** | 2 | 8 | | 16 | | >512 | 2 | 32 | >512 |
| **YN43** | **16** | 2 | 4 | | >512 | | >512 | 32 | 16 | >512 |
| **YN45** | **64** | 2 | 256 | | 8 | | 64 | 16 | >512 | >512 |
| **YN46** | **16** | 2 | 16 | | >512 | | >512 | 32 | 8 | >512 |
| **S1 Table(Continued)​** | | | | **MIC (μg/mL)​** | | | | | | |
| **NO.** | **COE-D8** | **NIT** | **FOS** | | **CEPH** | | **SUL** | **OFL** | **KM** | **AMP** |
| **Antimicrobial mechanisms** | Membrane disruption | Cell wall,DNA,RNA synthesis inhibitor | Cell wall synthesis inhibitor | | Cell wall synthesis inhibitor | | Competitive inhibitor of enzyme dihydropteroate synthetase | DNA gyrase inhibitor | Protein synthesis inhibitor | Cell wall synthesis inhibitor |
| **YN48** | **16** | 1 | 16 | | 8 | | >512 | 1 | 16 | >512 |
| **YN49** | **32** | 32 | 64 | | >512 | | >512 | 32 | 16 | >512 |
| **YN50** | **16** | 16 | 32 | | >512 | | >512 | 16 | 64 | >512 |
| **YN51** | **32** | 8 | 8 | | 8 | | 8 | 1 | 16 | >512 |
| **YN52** | **16** | 8 | 32 | | 4 | | 4 | 8 | 16 | >512 |
| **YN53** | **32** | 64 | 32 | | 32 | | 32 | 1 | 16 | >512 |
| **YN54** | **64** | 16 | 8 | | >512 | | >512 | 1 | 16 | >512 |
| **YN55** | **32** | 64 | 64 | | >512 | | >512 | 16 | 16 | >512 |
| **YN56** | **16** | 32 | 32 | | 8 | | 8 | 1 | 32 | 256 |
| **YN57** | **16** | 16 | 32 | | 32 | | 32 | 1 | 8 | 256 |
| **YN59** | **16** | 64 | 16 | | 16 | | 16 | 1 | 8 | 256 |
| **YN60** | **16** | 32 | 4 | | >512 | | >512 | 64 | 32 | >512 |
| **YN62** | **32** | 16 | 4 | | 16 | | >512 | 1 | 16 | >512 |
| **YN63** | **32** | 16 | 32 | | 8 | | 512 | 16 | 32 | >512 |
| **YN65** | **> 64** | 16 | 8 | | >512 | | 64 | 1 | 8 | >512 |
| **YN66** | **16** | 16 | 4 | | >512 | | >512 | 1 | 16 | >512 |
| **YN68** | **32** | 64 | 16 | | >512 | | 64 | 64 | 8 | >512 |
| **YN69** | **16** | 16 | 4 | | 8 | | >512 | 8 | 8 | >512 |
| **YN70** | **64** | 16 | 4 | | >512 | | 2 | 8 | 16 | >512 |
| **YN72** | **16** | 16 | 32 | | >512 | | 64 | 32 | 128 | >512 |
| **YN73** | **16** | 128 | >512 | | >512 | | >512 | 128 | 512 | >512 |
| **YN74** | **4** | 16 | 16 | | >512 | | >512 | 16 | 128 | >512 |
| **YN76** | **4** | 16 | 8 | | >512 | | 256 | 16 | 64 | >512 |
| **YN78** | **16** | 16 | 16 | | 32 | | >512 | 2 | 128 | >512 |
| **YN80** | **8** | 16 | 16 | | 4 | | >512 | 16 | >512 | >512 |
| **YN81** | **8** | 64 | 32 | | >512 | | >512 | 32 | >512 | >512 |
| **YN82** | **16** | 16 | 32 | | 32 | | >512 | 2 | 128 | >512 |
| **YN83** | **16** | 16 | 16 | | >512 | | >512 | 32 | 64 | >512 |
| **YN84** | **16** | 16 | 16 | | 8 | | >512 | 1 | 64 | >512 |
| **YN85** | **8** | 32 | 4 | | 16 | | >512 | 8 | 8 | >512 |
| **YN86** | **16** | 32 | 32 | | 32 | | >512 | 1 | 8 | 16 |
| **YN87** | **16** | 32 | 16 | | >512 | | >512 | 8 | 16 | >512 |
| **YN88** | **16** | 128 | 32 | | >512 | | >512 | 64 | 16 | >512 |
| **YN89** | **16** | 32 | >512 | | >512 | | >512 | 32 | 16 | >512 |
| **YN90** | **64** | >512 | 16 | | >512 | | >512 | 64 | 8 | >512 |
| **YN91** | **16** | >512 | 16 | | >512 | | >512 | 1 | 8 | >512 |
| **YN92** | **16** | 32 | 8 | | >512 | | >512 | 16 | 64 | >512 |
| **YN93** | **8** | 16 | 32 | | 128 | | >512 | 1 | 8 | >512 |
| **YN94** | **16** | 16 | 32 | | 16 | | >512 | 16 | 8 | 8 |
| **YN95** | **16** | 32 | 64 | | >512 | | >512 | 64 | 128 | >512 |
| **YN96** | **8** | 32 | 16 | | 64 | | 64 | 1 | 64 | >512 |
| **YN97** | **16** | 32 | 32 | | 16 | | >512 | 8 | 32 | >512 |
| **S1 Table(Continued)​** | | | | | | **MIC (μg/mL)​** | | | | |
| **NO.** | **COE-D8** | **NIT** | **FOS** | | **CEPH** | | **SUL** | **OFL** | **KM** | **AMP** |
| **Antimicrobial mechanisms** | Membrane disruption | Cell wall,DNA,RNA synthesis inhibitor | Cell wall synthesis inhibitor | | Cell wall synthesis inhibitor | | Competitive inhibitor of enzyme dihydropteroate synthetase | DNA gyrase inhibitor | Protein synthesis inhibitor | Cell wall synthesis inhibitor |
| **YN98** | **16** | 32 | 64 | | 128 | | >512 | 4 | 32 | >512 |
| **YN99** | **16** | 16 | 64 | | 8 | | >512 | 1 | 8 | 8 |
| **YN100** | **16** | 32 | 16 | | 32 | | >512 | 1 | 8 | >512 |
| **YN101** | **32** | 32 | 32 | | 8 | | >512 | 1 | 8 | >512 |
| **YN102** | **32** | 16 | 8 | | >512 | | >512 | 16 | >512 | >512 |
| **YN103** | **16** | 32 | 16 | | >512 | | >512 | 8 | >512 | >512 |
| **YN105** | **16** | >512 | 16 | | >512 | | >512 | 16 | 16 | >512 |
| **YN106** | **16** | >512 | 32 | | >512 | | >512 | 16 | 16 | >512 |
| **YN107** | **8** | 32 | 8 | | 32 | | >512 | 8 | 512 | >512 |
| **YN108** | **8** | 16 | 16 | | 64 | | 32 | 1 | 32 | >512 |
| **YN110** | **16** | 32 | 16 | | >512 | | >512 | 16 | 128 | >512 |
| **YN111** | **16** | 16 | 128 | | >512 | | >512 | 1 | 8 | >512 |
| **YN112** | **16** | 32 | 16 | | 8 | | >512 | 16 | 16 | >512 |
| **YN113** | **16** | 32 | 16 | | 8 | | >512 | 32 | 8 | 256 |
