# Supplementary material for: A novel COE-D8-fosfomycin conjugate effectively combats first-line antibiotic-resistant uropathogenic Escherichia coli
Source: PLoS One. 2026 Jul 8;21(7):e0352997. doi: 10.1371/journal.pone.0352997 (PMC13345249; doi:10.1371/journal.pone.0352997)
Supplement: S2 Table — All single base substitutions were non-synonymous mutations coding for either an amino acid substitution.The last column shows the gene before resistance evolution (left of right arrow) mutate to the gene on the right hand side experiencing corresponding evolution day. (DOCX) [file pone.0352997.s004.docx]

**S2 Table. Non-synonymous single-base substitutions acquired during *in vitro* resistance evolution.**

| **Strains** | **Genes** | **Mutations during resistance evolution** |
| --- | --- | --- |
| Strain 63 | efflux RND transporter permease *acrB* | 515W->stop codon |
| Strain 63 | *nlpD* putative outer membrane lipoprotein | fragment loss from 368 to 380 |
| Strain 63 | *mzrA* | 70P->T |
| Strain 63 | *malT* transcriptional activator | 319C->Y |
| Strain 74 | multidrug efflux transporter *emrE* | 3L->P |
| Strain 74 | *malT* transcriptional activator | 439S->I |
| Strain 76 | *ribB* (3,4-dihydroxy-2-butanone 4-phosphate synthase) | 100D->E |
