# Supplementary material for: A novel COE-D8-fosfomycin conjugate effectively combats first-line antibiotic-resistant uropathogenic Escherichia coli
Source: PLoS One. 2026 Jul 8;21(7):e0352997. doi: 10.1371/journal.pone.0352997 (PMC13345249; doi:10.1371/journal.pone.0352997)
Supplement: S3 Table — The gene of MzrA shows a lower docking energy of −5.0 kcal/mol than docking with the protein before resistance evolution −4.6 kcal/mol. There are no docking energy change before and after resistance evolution among the gene of MalT, EmrE and RibB. (DOCX) [file pone.0352997.s005.docx]

**S3 Table.** **Docking Affinity in Autodock Vina.**

| **Gene** | **Ligand** | **Affinity (kcal/mol)** |
| --- | --- | --- |
| MzrA^1^ | COE-D8 | -5 |
| MzrA^2^ |  | -4.6 |
| MalT transcriptional activator^1^ |  | -7.1 |
| MalT transcriptional activator^2^ |  | -7.2 |
| multidrug efflux transporter EmrE^1^ |  | -4.2 |
| multidrug efflux transporter EmrE^2^ |  | -4.1 |
| RibB (3,4-dihydroxy-2-butanone 4-phosphate synthase)^1^ |  | -4.5 |
| RibB (3,4-dihydroxy-2-butanone 4-phosphate synthase)^2^ |  | -4.5 |
