# Supplementary material for: A novel COE-D8-fosfomycin conjugate effectively combats first-line antibiotic-resistant uropathogenic Escherichia coli
Source: PLoS One. 2026 Jul 8;21(7):e0352997. doi: 10.1371/journal.pone.0352997 (PMC13345249; doi:10.1371/journal.pone.0352997)
Supplement: S4 Table — Data are expressed as the mean ± standard deviation (SD) (n = 5 per group). Statistical significance between Day 0 and Day 7 was determined using a two-tailed Student’s t-test (P < 0.05). Reference standard ranges for healthy mice: AST (36.31–235.48 U/L), ALT (10.06–96.47 U/L), and BUN (10.81–34.74 mg/dL). Abbreviations: AST, aspartate aminotransferase; ALT, alanine aminotransferase; BUN, blood urea nitrogen. Statistical analysis was performed using Student’s t-test; differences are considered statistically significant with probability P < 0.05. (DOCX) [file pone.0352997.s006.docx]

**S4 Table. *In vivo* safety evaluation of COE-D8, fosfomycin, and their combination following intraperitoneal injection in mice.**

|  | **AST (U/L)** | |  | **ALT (U/L)** | |  | **BUN ( mg/dl )** | |
| --- | --- | --- | --- | --- | --- | --- | --- | --- |
|  | **0 day** | **7 day** |  | **0 day** | **7 day** |  | **0 day** | **7 day** |
| **COE-D8 25mg /kg** | 108.22 | 356.55 |  | 15.75 | 180.3 |  | 22.70 | 28.64 |
| **FOS 50mg /kg** | 121.05 | 79.38 |  | 17.83 | 23.19 |  | 23.09 | 20.71 |
| **COE-D8 25mg/kg+FOS 50 mg/kg** | 103.22 | 227.30 |  | 8.26 | 36.83 |  | 19.00 | 17.20 |
