# Supplementary material for: A novel COE-D8-fosfomycin conjugate effectively combats first-line antibiotic-resistant uropathogenic Escherichia coli
Source: PLoS One. 2026 Jul 8;21(7):e0352997. doi: 10.1371/journal.pone.0352997 (PMC13345249; doi:10.1371/journal.pone.0352997)
Supplement: Attachment — Submitted filename: Response to Reviewers.docx [file pone.0352997.s008.docx]

Reviewers' comments:

Reviewer's Responses to Questions

Comments to the Author

5. Review Comments to the Author

Please use the space provided to explain your answers to the questions above. You may also include additional comments for the author, including concerns about dual publication, research ethics, or publication ethics. (Please upload your review as an attachment if it exceeds 20,000 characters)

Reviewer #1: Lin Ruan and colleagues investigate the antimicrobial efficacy of a novel membrane-intercalating compound, COE-D8, against multidrug-resistant uropathogenic Escherichia coli (UPEC). They show that COE-D8 exhibits potent bactericidal activity, outperforming several first-line antibiotics, with MICs ≤32 μg/mL for over 90% of 93 clinical UPEC isolates. Mechanistic assays reveal that COE-D8 disrupts bacterial membranes, although its cytotoxicity limits standalone clinical use. To address this, the authors combine COE-D8 with fosfomycin, which significantly reduces cytotoxicity in mammalian cells and improves survival in treated mice. Whole-genome sequencing and molecular docking suggest that mutations in membrane-associated proteins, including MzrA, may contribute to the development of resistance. These findings support the COE-D8/fosfomycin combination as a promising strategy against antibiotic-resistant UPEC.

The manuscript addresses an important medical challenge and is supported by a robust experimental data set, including in vitro assays, in vivo toxicity studies, and genome-wide bioinformatics. However, several revisions are needed to improve the manuscript's clarity and compliance with publication standards.

1/ Language and Grammar

The manuscript contains numerous grammatical, syntactic, and stylistic issues that hinder comprehension.

Please revise overly long or awkward sentences such as:

“The experiment described above did not differentiate whether COE-D8 acts to inhibit bacterial growth or to kill bacteria.”

proposition: “The previous experiment did not determine whether COE-D8 was bacteriostatic or bactericidal.”

Avoid overly cautious phrasing such as:

“This suggests that there is a significant likelihood that patients may not respond…”

proposition: “This suggests that many patients may not respond to treatment.”

I have revised some awkward sentences as per your instructions and translated them into English.

2/ Is COE-D8 patented? Has it been approved as a therapeutic agent in any country? This should be clearly stated in the introduction or discussion.

I have added it to the discussion section as per your instructions：It is important to note that COE-D8 is currently not patented and has not been approved as a therapeutic agent in any country. Nevertheless, its promising antimicrobial properties warrant further investigation.

3/ Some figures lack information regarding statistical tests and significance indicators. Please standardize this across all figures and legends, specifying p-values, error bars, and the statistical methods used.有些图表缺少有关统计检验和显著性指标的信息。请在所有图表及图例中统一规范这一内容，明确标注 p 值、误差范围以及所采用的统计方法。

I have annotated figures 5c to 5g according to your directions.

4/ (p13) The study involves animal experimentation. A dedicated ethics sub-section should be added to the Methods section with confirmation that all procedures followed current animal welfare regulations and guidelines.

It has been incorporated into Methods section 2.4.6

5/ The term “Luria-Bertani” should be corrected to the more accurate name Lysogeny Broth (see: Bertani, J. Bacteriol. 2004).

The amendments have been made according to your directions.

6/ (P.20) The Data Availability Statement currently indicates “data available on request.” In accordance with PLOS ONE policy, please deposit all genome sequence data and raw data in a public repository (e.g., NCBI SRA, Dryad, Zenodo) and include accession numbers in the manuscript.

It has been added to Method 2.3.10

7/ (P.21) Were the 93 UPEC isolates derived from 93 independent patients, or were multiple isolates obtained from the same patient at different time points?

It has been added to Result 3.1 It is important to clarify that each of these 93 isolates was derived from an independent patient, ensuring a genetically diverse cohort for analysis. The genomes of these isolates were subjected to next-generation sequencing, and a phylogenetic tree was constructed (S1 Fig.), confirming their distinct lineages.

8/ You propose MzrA as a resistance-associated target. Were gene expression levels of mzrA or related pathways assessed following COE-D8 exposure?

We thank the reviewer for raising this insightful question regarding the potential role of mzrA expression levels in the observed resistance. We agree that investigating transcriptional changes following COE-D8 exposure would be a valuable endeavor.

However, we are currently unable to perform the suggested analyses due to the following constraints:

1. Regarding pathway analysis: Reliable pathway analysis requires a highly annotated reference genome, such as E. coli K-12 MG1655. As shown in Appendix Fig.1, our clinical strains (belonging to phylogroups B1 and B2) are evolutionarily distant from the K-12 strain (phylogroup A), making reliable comparative pathway analysis unfeasible.

2. Practical limitations for gene expression analysis: Conducting qPCR experiments would require re-synthesizing COE-D8 and applying it as a stressor to specific frozen bacterial stocks (from day 0 and day 30). This is currently impractical, as the corresponding author has relocated abroad, and access to the original laboratory's specific bacterial strains and the key personnel involved is no longer available.

Despite these limitations, we have carefully considered the potential outcomes of such expression studies. It is plausible that the expression level of *mzrA* might not change significantly. Our proposed resistance mechanism is primarily based on a structural alteration in the MzrA protein (Thr70Pro). Molecular docking studies suggest this mutation directly affects COE-D8 binding affinity. This model of mutation-driven resistance, rather than regulation of gene expression, is strongly supported by several lines of evidence:

1.Phenotypic Resistance: A substantial (4- to 128-fold) increase in MIC after long-term COE-D8 exposure provides clear evidence of an adaptive response.

2.Specific Genomic Alteration: Whole-genome sequencing identified a single, non-synonymous mutation (Thr70Pro) in the mzrA gene of the evolved strains.

3.Energetic Validation: Molecular docking indicates this mutation is located within the predicted COE-D8 binding pocket and is associated with an altered binding energy, providing a mechanistic rationale for the reduced susceptibility.

4.Literature Support: MzrA's established role in maintaining cell envelope integrity aligns perfectly with the need to counteract COE-D8's membrane-disrupting action, making it a biologically relevant target for adaptive mutations.

Therefore, although expression data are not available, the convergent evidence strongly suggests that the acquired resistance is likely mediated by a structural mutation in MzrA rather than transcriptional regulation.

In response to this valuable comment, we have proactively added a paragraph to the Discussion section to acknowledge this limitation: "Among these, a specific Thr70Pro mutation in the MzrA protein emerged as a key finding. It should be acknowledged that a limitation of this study is the lack of gene expression analysis of mzrA under COE-D8 exposure. Such experiments could further elucidate whether resistance is mediated solely by the observed Thr70Pro mutation or also involves changes in gene expression regulation. Nevertheless, the convergence of phenotypic resistance, a specific amino acid alteration, and docking data provides a multi-faceted argument for the role of MzrA in UPEC's adaptive resistance to COE-D8. Validating the functional impact of this mutation remains an important objective for future research."

Reviewer #2: The authors have taken an innovative approach to addressing antibiotic resistance. While the manuscript is generally sound, a few revisions are necessary to improve readability before it can be finalized.

1.In the abstract, there should not be line break. Please change it according to the journal criteria.

Eliminated

2.At the end of the introduction, the author mentioned their findings in a brief. But it would be better to add the objectives of this study which is missing.

It has been added to the last paragraph of the introduction：The objectives of this study were to investigate the molecular mechanisms of UPEC resistanceand to evaluate the biocompatibility of our therapeutic approach in an animal model, thereby laying the groundwork for future in vivo bactericidal experiments.

Materials and methods:

3. It is mentioned with the reference of COE-D8 synthesized but add in a brief of this protocol easily understandable.

It has been added to Method 2.3.1

4. The author did not included statistical analysis part which is supposed to be added with reference software the author used.

It has been added in fig5. c-g

5. Please ad relevant references in the materials and methods section. References are missing in bioinformatics section

They have been added.

Result:

6. section 3.1 seems combination of your findings and remarks. However, it is supposed to present only the findings from your study and the conclusion marks should be in the discussion part.

It has been modified

7. Figure 1c is cited in the result section and the legend of the figure consists the statistical description and the test name. But it should be included in the description of the result as well and the outcome of test. Please reduce the statements from the 3.1 section and the test findings with proper figure citation.

It has been modified

7. In 3.2 section, the author described reduction of bacterial concentration but the title is related to phylogeny which is missing in the description in this section.

It has been modified

8. Please chnage the whole result section with just your findings. Remarks should be in the discussion part.

It has been modified

10. Please submit your revised manuscript after following the guidelines given by the journal to arrange the information in systematic waym such as reference style, font, arrangment of table, and others.

It has been modified

Reviewer #3: This manuscript presents a study on the antimicrobial potential of a conjugated oligoelectrolyte, COE-D8, and its synergistic combination with fosfomycin against multidrug-resistant uropathogenic Escherichia coli (UPEC). The authors provide a methodologically sound investigation, including in vitro antibacterial efficacy, cytotoxicity evaluation, resistance evolution, mechanistic insights via docking simulations, and in vivo toxicity assessment. The study is timely, addresses a pressing clinical issue, and could contribute significantly to the development of novel antimicrobial strategies.

However, there are some concerns regarding experimental clarity, data interpretation, and presentation that need to be addressed before publication.

1. The docking simulation showed that the binding energy of COE-D8 to the mutant MzrA protein increased by only 0.4 kcal/mol compared to the wild-type. Given the limited precision of docking tools such as AutoDock Vina, this small energy difference may not be sufficient to substantiate MzrA as a key resistance determinant. The authors should clarify whether this minor difference is biologically meaningful and whether additional evidence supports MzrA’s involvement.

We sincerely thank the reviewer for this insightful comment regarding the precision of docking tools and the biological significance of the small change in binding energy (ΔG = +0.4 kcal/mol). The reviewer rightly points out a crucial limitation of computational methods.

We acknowledge that docking simulations with tools like AutoDock Vina have inherent precision limits, and a ΔG of +0.4 kcal/mol alone may not be considered statistically significant. However, we wish to clarify that our conclusion regarding the role of the MzrA Thr70Pro mutation is not based solely on this docking energy difference. Instead, it is supported by a convergence of multiple, independent lines of evidence presented in the manuscript, which together build a compelling case:

Genetic-Phenotypic Correlation: The Thr70Pro mutation was specifically identified and repeatedly observed in UPEC strains that evolved resistance to COE-D8 through experimental evolution. This provides a direct genetic link to the resistant phenotype.

Biological Plausibility: MzrA is a membrane-associated regulator of cell wall synthesis. Our conclusion states that "the potent mechanism of COE-D8 against UPEC is associated with the cell wall protein... MzrA." It is mechanistically plausible that a mutation in a protein responsible for maintaining cell wall integrity could confer resistance to a compound like COE-D8, which we hypothesize may cause membrane disruption.

Exclusion of Other Direct Targets: Our bioinformatic analysis revealed mutations in other proteins (e.g., NlpD, MalT, EmrE). However, docking studies indicated that their mutation sites were not located within the predicted binding pocket for COE-D8. This contrast makes the MzrA mutation, which is predicted to be near the binding site, stand out as a more direct candidate related to drug-target interaction.

Multi-faceted Argument: As we stated in the Discussion, "the convergence of phenotypic resistance, a specific amino acid alteration, and docking data provides a multi-faceted argument for the role of MzrA in UPEC's adaptive resistance to COE-D8." The docking result, while small, is consistent with the overall narrative and adds a computational dimension to the genetic and phenotypic findings.

We agree with the reviewer that functional validation is the next critical step. We have explicitly acknowledged this as a limitation and a goal for future research: "Validating the functional impact of this mutation remains an important objective for future research."

In summary, we present the docking data not as standalone proof, but as one piece of a cohesive puzzle. We will revise the manuscript to more clearly emphasize that the biological significance is derived from the collective weight of the evidence rather than the modest docking energy change alone.

2. Apart from MzrA, other genes listed in Table S2 were also found mutated, but their roles in resistance were not evaluated or discussed in sufficient detail. Could the authors speculate or provide functional predictions regarding how these genes might contribute to COE-D8 resistance? For example, a pathway-based discussion would be valuable.

We thank for this insightful comment and valuable suggestion. We agree that discussing the potential roles of other mutated genes would strengthen the discussion of our findings. Accordingly, we have provided functional predictions for the key mutated genes mentioned by the supporting information:

1.MalT: As a master regulator of carbon metabolism, mutations in MalT could lead to altered expression of its regulon members, including porins and transporters, potentially reducing membrane permeability to COE-D8. Furthermore, given the link between carbon metabolism and biofilm formation, MalT may indirectly contribute to resistance by enhancing biofilm formation.

2.EmrE: This gene encodes a small multidrug efflux pump. Mutations in EmrE could enhance its ability to expel COE-D8 from the cell using the proton motive force, a well-established antibiotic resistance mechanism (Victor Ovchinnikov., 2018, PNAS).

3.ribB (encoding 3,4-dihydroxy-2-butanone 4-phosphate synthase): Mutations in this enzyme, which is involved in essential cofactor synthesis, could enhance bacterial fitness and adaptability under antibiotic stress by sustaining metabolic homeostasis.

In response to this valuable comment, we have proactively added a paragraph to the Discussion section:

However, we still speculate that for MalT, altered regulation of its target genes—including those for porins and transporters—could reduce membrane permeability to COE-D8. MalT may also indirectly promote biofilm formation, a known resistance mechanism. The mutation in emrE is significant as it encodes a multidrug efflux pump that expels drugs using the proton motive force (Che et al., 2004, PNAS). Finally, a mutation in ribB, involved in essential cofactor synthesis, could enhance bacterial fitness under antibiotic stress by sustaining metabolic homeostasis. Thus, while functional validation is needed, these mutations highlight potential adaptive strategies involving membrane integrity, efflux, and metabolic regulation, which may operate independently of direct drug-target interaction.

3. The combination of fosfomycin with COE-D8 significantly reduced cytotoxicity in 3T3 cells. How might fosfomycin mitigate the cytotoxicity of COE-D8? This deserves further explanation in the paper. Moreover, the IC50 of 16 μg/mL is still relatively toxic, especially considering the MIC range for UPEC. Have the authors considered chemical modifications of COE-D8 to improve its selectivity and reduce mammalian toxicity?

Regarding the reviewer's insightful question on how fosfomycin mitigates the cytotoxicity of COE-D8, we propose that this effect is likely indirect and results from synergistic antibacterial action. Fosfomycin, by inhibiting bacterial cell wall synthesis, compromises the integrity of the bacterial envelope. This damage likely potentiates the membrane-disrupting activity of COE-D8, allowing the latter to achieve potent bactericidal efficacy at a lower concentration. This hypothesis is consistent with our time-kill assay observations, where the combination resulted in rapid killing of UPEC. Given that the cytotoxicity of COE-D8 is dose-dependent, as indicated by the MTT assay, reducing its effective dose through combination with fosfomycin would consequently diminish its adverse effects on mammalian cells. This explanation aligns well with our in vivo findings of reduced organ damage in mouse models, and we have incorporated this discussion into the revised manuscript：

We hypothesize that this reduction in cytotoxicity stems from a synergistic antibacterial action. Fosfomycin, by inhibiting bacterial cell wall synthesis, compromises the integrity of the bacterial envelope. This damage likely potentiates the membrane-disrupting activity of COE-D8, allowing it to achieve potent bactericidal efficacy at a lower concentration—a phenomenon supported by our time-kill assays. Given the dose-dependent cytotoxicity of COE-D8 (as indicated by MTT assay), reducing its effective dose in combination with Fosfomycin would consequently diminish its adverse effects on mammalian cells.

Chemical modification of COE-D8 should indeed be considered to improve its selectivity and reduce mammalian toxicity, which aligns with the statement we included in the discussion: "need for further chemical modifications to reduce toxicity and enhance efficacy." We recognize that the characteristic long, hydrophobic backbone of COE molecules enables them to insert into and stabilize lipid bilayers. We anticipate that through rational structural optimization, such as fine-tuning the amphiphilic balance, we can precisely modulate the strength of COE-membrane interactions with different cell types. This strategy holds promise for enhancing their targeting specificity towards bacterial membranes—which differ in lipid composition and physical properties from their mammalian counterparts—thereby achieving more effective membrane perturbation while mitigating toxicity to mammalian cells.

We have consolidated this perspective and used it to elaborate in greater detail on the originally brief mention in the Discussion section：

For instance, rational optimization could focus on fine-tuning the amphiphilicity of the long hydrophobic COE backbone, which is responsible for membrane insertion and stabilization, to preferentially target bacterial over mammalian membranes.

4. While the authors assessed the in vivo toxicity of COE-D8, the manuscript lacks evaluation of its in vivo antimicrobial efficacy. Demonstrating bacterial burden reduction in infected animals would greatly strengthen the translational value of the study. Please consider including such data or discussing this limitation explicitly.

Thank you for this valuable comment. We fully agree that in vivo antimicrobial efficacy data would significantly strengthen the translational value of COE-D8. However, conducting such an experiment at this stage presents practical challenges for two main reasons: First, we are currently pursuing chemical optimization of COE-D8 to reduce its in vitro toxicity while maintaining or improving antibacterial activity, and we hope to present corresponding in vivo results with an improved analog in future studies. Second, as I have left the affiliation listed in the manuscript and the corresponding author is currently overseas, reapplying for animal experiment licenses and regaining access to necessary resources would be difficult. We will explicitly acknowledge this limitation in the discussion section and highlight it as an important objective for future work：

Furthermore, the in vivo antimicrobial efficacy of COE-D8 remains to be evaluated, which will be a critical focus for future studies on optimized analogs.
