# Supplementary material for: A novel COE-D8-fosfomycin conjugate effectively combats first-line antibiotic-resistant uropathogenic Escherichia coli
Source: PLoS One. 2026 Jul 8;21(7):e0352997. doi: 10.1371/journal.pone.0352997 (PMC13345249; doi:10.1371/journal.pone.0352997)
Supplement: Attachment — Submitted filename: 20260325_submit_Response to Reviewers.docx [file pone.0352997.s009.docx]

Review Comments to the Author

Please use the space provided to explain your answers to the questions above. You may also include additional comments for the author, including concerns about dual publication, research ethics, or publication ethics. (Please upload your review as an attachment if it exceeds 20,000 characters)

Reviewer #1: On page 61: mzrA should be italicized in the sentence, “It should be acknowledged that a limitation of this study is the lack of gene expression analysis of mzrA.”

**Author Response**: We sincerely appreciate your professional and detailed comment on our manuscript. We have fully revised the manuscript in strict accordance with your suggestion: the gene name mzrA in the corresponding sentence on page 61 has been formatted in italics, which fully complies with the gene nomenclature guidelines of PLOS ONE.Meanwhile, we have highlighted this modification in yellow in the Revised Manuscript with Track Changes for your convenient review and verification. In addition, taking this format requirement as the standard, we have conducted a comprehensive cross-check of the writing format of all gene names and species scientific names throughout the manuscript, to ensure the uniform format of the full text and full compliance with the journal's submission formatting specifications.

Reviewer #2: The authors have adequately addressed all of my comments and concerns. The revisions have improved the clarity and scientific rigor of the manuscript. I find the responses satisfactory, and the manuscript entitled “A Novel COE-D8-Fosfomycin Conjugate Effectively Against First-Line Antibiotic-Resistant Uropathogenic Escherichia coli” is now acceptable in its current form

**Author Response:** We sincerely thank you for your recognition of our research work, as well as all the professional comments and valuable suggestions you have put forward during the review process. Your suggestions have greatly helped us improve the content of the manuscript, and enhance the clarity of presentation and scientific rigor of this study.We have carefully reviewed all of your review comments, and re-checked the research content, data presentation and narrative expression of the manuscript in an all-round way, to ensure the rigor, standardization and integrity of the manuscript content. We once again sincerely appreciate your valuable time and effort dedicated to the review of our manuscript.

Reviewer #3: (No Response)
**Author Response:** We have carefully checked all the review feedback from Reviewer #3, and there are no comments or suggestions requiring revision. We sincerely appreciate the time and effort that Reviewer #3 has dedicated to the review of our manuscript.
