# Supplementary material for: A novel COE-D8-fosfomycin conjugate effectively combats first-line antibiotic-resistant uropathogenic Escherichia coli
Source: PLoS One. 2026 Jul 8;21(7):e0352997. doi: 10.1371/journal.pone.0352997 (PMC13345249; doi:10.1371/journal.pone.0352997)
Supplement: Peer review history

# Peer review: Decision Letter 0

**DOI:** 10.1371/journal.pone.0352997.r001

**Authors:** Eva Torres-Sangiao

13 Aug 2025

PONE-D-25-19542

A Novel COE-D8-Fosfomycin Conjugate Effectively Against First-line Antibiotic Resistant Uropathogenic *Escherichia coli*

PLOS ONE

Dear Dr. Ruan,

Thank you for submitting your manuscript to PLOS ONE. After careful consideration, we feel that it has merit but does not fully meet PLOS ONE’s publication criteria as it currently stands. Therefore, we invite you to submit a revised version of the manuscript that addresses the points raised during the review process.

Please submit your revised manuscript by Sep 27 2025 11:59PM. If you will need more time than this to complete your revisions, please reply to this message or contact the journal office at plosone@plos.org. When you're ready to submit your revision, log on to https://www.editorialmanager.com/pone/ and select the 'Submissions Needing Revision' folder to locate your manuscript file.

Please include the following items when submitting your revised manuscript:

- A rebuttal letter that responds to each point raised by the academic editor and reviewer(s). You should upload this letter as a separate file labeled 'Response to Reviewers'.
- A marked-up copy of your manuscript that highlights changes made to the original version. You should upload this as a separate file labeled 'Revised Manuscript with Track Changes'.
- An unmarked version of your revised paper without tracked changes. You should upload this as a separate file labeled 'Manuscript'.

-->If you would like to make changes to your financial disclosure, please include your updated statement in your cover letter. Guidelines for resubmitting your figure files are available below the reviewer comments at the end of this letter.

If applicable, we recommend that you deposit your laboratory protocols in protocols.io to enhance the reproducibility of your results. Protocols.io assigns your protocol its own identifier (DOI) so that it can be cited independently in the future. For instructions see: https://journals.plos.org/plosone/s/submission-guidelines#loc-laboratory-protocols. Additionally, PLOS ONE offers an option for publishing peer-reviewed Lab Protocol articles, which describe protocols hosted on protocols.io. Read more information on sharing protocols at https://plos.org/protocols?utm_medium=editorial-email&utm_source=authorletters&utm_campaign=protocols.

We look forward to receiving your revised manuscript.

Kind regards,

Eva Torres-Sangiao, PhD

Academic Editor

PLOS ONE

Journal requirements:

When submitting your revision, we need you to address these additional requirements.

1. Please ensure that your manuscript meets PLOS ONE's style requirements, including those for file naming. The PLOS ONE style templates can be found at

https://journals.plos.org/plosone/s/file?id=wjVg/PLOSOne_formatting_sample_main_body.pdf and

https://journals.plos.org/plosone/s/file?id=ba62/PLOSOne_formatting_sample_title_authors_affiliations.pdf

2. Thank you for stating the following financial disclosure:

“This work was supported by the National Natural Science Foundation of China No.32070818 and 82460142 to C.M.G., Natural Science Foundation of Yunnan Province of China [202001BB050005], the Xingdian talent support program of Yunnan Province to C.M.G., and Guangdong Hybribio Biotech Funding No.H20230314, H20230311 and H20230313 to C.M.G.”

Please state what role the funders took in the study.  If the funders had no role, please state: "The funders had no role in study design, data collection and analysis, decision to publish, or preparation of the manuscript."

If this statement is not correct you must amend it as needed.

Please include this amended Role of Funder statement in your cover letter; we will change the online submission form on your behalf.

3. Thank you for stating the following in the Acknowledgments Section of your manuscript:

“This work was supported by the National Natural Science Foundation of China No.32070818 and 82460142 to C.M.G., Natural Science Foundation of Yunnan Province of China [202001BB050005], the Xingdian talent support program of Yunnan Province to C.M.G., and Guangdong Hybribio Biotech Funding No.H20230314, H20230311 and H20230313 to C.M.G.”

We note that you have provided funding information that is currently declared in your Funding Statement. However, funding information should not appear in the Acknowledgments section or other areas of your manuscript. We will only publish funding information present in the Funding Statement section of the online submission form.

Please remove any funding-related text from the manuscript and let us know how you would like to update your Funding Statement. Currently, your Funding Statement reads as follows:

“This work was supported by the National Natural Science Foundation of China No.32070818 and 82460142 to C.M.G., Natural Science Foundation of Yunnan Province of China [202001BB050005], the Xingdian talent support program of Yunnan Province to C.M.G., and Guangdong Hybribio Biotech Funding No.H20230314, H20230311 and H20230313 to C.M.G.”

Please include your amended statements within your cover letter; we will change the online submission form on your behalf.

4. In the online submission form, you indicated that [Insert text from online submission form here].

All PLOS journals now require all data underlying the findings described in their manuscript to be freely available to other researchers, either 1. In a public repository, 2. Within the manuscript itself, or 3. Uploaded as supplementary information.

This policy applies to all data except where public deposition would breach compliance with the protocol approved by your research ethics board. If your data cannot be made publicly available for ethical or legal reasons (e.g., public availability would compromise patient privacy), please explain your reasons on resubmission and your exemption request will be escalated for approval.

5. We note that you have indicated that there are restrictions to data sharing for this study. PLOS only allows data to be available upon request if there are legal or ethical restrictions on sharing data publicly. For more information on unacceptable data access restrictions, please see http://journals.plos.org/plosone/s/data-availability#loc-unacceptable-data-access-restrictions.

Before we proceed with your manuscript, please address the following prompts:

a) If there are ethical or legal restrictions on sharing a de-identified data set, please explain them in detail (e.g., data contain potentially identifying or sensitive patient information, data are owned by a third-party organization, etc.) and who has imposed them (e.g., a Research Ethics Committee or Institutional Review Board, etc.). Please also provide contact information for a data access committee, ethics committee, or other institutional body to which data requests may be sent.

b) If there are no restrictions, please upload the minimal anonymized data set necessary to replicate your study findings to a stable, public repository and provide us with the relevant URLs, DOIs, or accession numbers. For a list of recommended repositories, please see

https://journals.plos.org/plosone/s/recommended-repositories. You also have the option of uploading the data as Supporting Information files, but we would recommend depositing data directly to a data repository if possible.

We will update your Data Availability statement on your behalf to reflect the information you provide.

5. Please include captions for your Supporting Information files at the end of your manuscript, and update any in-text citations to match accordingly. Please see our Supporting Information guidelines for more information: http://journals.plos.org/plosone/s/supporting-information.

If the reviewer comments include a recommendation to cite specific previously published works, please review and evaluate these publications to determine whether they are relevant and should be cited. There is no requirement to cite these works unless the editor has indicated otherwise.

Please review your reference list to ensure that it is complete and correct. If you have cited papers that have been retracted, please include the rationale for doing so in the manuscript text, or remove these references and replace them with relevant current references. Any changes to the reference list should be mentioned in the rebuttal letter that accompanies your revised manuscript. If you need to cite a retracted article, indicate the article’s retracted status in the References list and also include a citation and full reference for the retraction notice.

[Note: HTML markup is below. Please do not edit.]

Reviewers' comments:

Reviewer's Responses to Questions

**Comments to the Author**

1. Is the manuscript technically sound, and do the data support the conclusions?

The manuscript must describe a technically sound piece of scientific research with data that supports the conclusions. Experiments must have been conducted rigorously, with appropriate controls, replication, and sample sizes. The conclusions must be drawn appropriately based on the data presented.

Reviewer #1: Yes

Reviewer #2: Yes

Reviewer #3: Yes

**********

2. Has the statistical analysis been performed appropriately and rigorously?

Reviewer #1: I Don't Know

Reviewer #2: No

Reviewer #3: Yes

**********

3. Have the authors made all data underlying the findings in their manuscript fully available?

The PLOS Data policy requires authors to make all data underlying the findings described in their manuscript fully available without restriction, with rare exception (please refer to the Data Availability Statement in the manuscript PDF file). The data should be provided as part of the manuscript or its supporting information, or deposited to a public repository. For example, in addition to summary statistics, the data points behind means, medians and variance measures should be available. If there are restrictions on publicly sharing data—e.g. participant privacy or use of data from a third party—those must be specified.

Reviewer #1: No

Reviewer #2: Yes

Reviewer #3: Yes

**********

4. Is the manuscript presented in an intelligible fashion and written in standard English?

PLOS ONE does not copyedit accepted manuscripts, so the language in submitted articles must be clear, correct, and unambiguous. Any typographical or grammatical errors should be corrected at revision, so please note any specific errors here.

Reviewer #1: Yes

Reviewer #2: Yes

Reviewer #3: Yes

**********

5. Review Comments to the Author

Please use the space provided to explain your answers to the questions above. You may also include additional comments for the author, including concerns about dual publication, research ethics, or publication ethics. (Please upload your review as an attachment if it exceeds 20,000 characters)

Reviewer #1: Lin Ruan and colleagues investigate the antimicrobial efficacy of a novel membrane-intercalating compound, COE-D8, against multidrug-resistant uropathogenic Escherichia coli (UPEC). They show that COE-D8 exhibits potent bactericidal activity, outperforming several first-line antibiotics, with MICs ≤32 μg/mL for over 90% of 93 clinical UPEC isolates. Mechanistic assays reveal that COE-D8 disrupts bacterial membranes, although its cytotoxicity limits standalone clinical use. To address this, the authors combine COE-D8 with fosfomycin, which significantly reduces cytotoxicity in mammalian cells and improves survival in treated mice. Whole-genome sequencing and molecular docking suggest that mutations in membrane-associated proteins, including MzrA, may contribute to the development of resistance. These findings support the COE-D8/fosfomycin combination as a promising strategy against antibiotic-resistant UPEC.

The manuscript addresses an important medical challenge and is supported by a robust experimental data set, including in vitro assays, in vivo toxicity studies, and genome-wide bioinformatics. However, several revisions are needed to improve the manuscript's clarity and compliance with publication standards.

1/ Language and Grammar

The manuscript contains numerous grammatical, syntactic, and stylistic issues that hinder comprehension.

Please revise overly long or awkward sentences such as:

“The experiment described above did not differentiate whether COE-D8 acts to inhibit bacterial growth or to kill bacteria.”

proposition: “The previous experiment did not determine whether COE-D8 was bacteriostatic or bactericidal.”

Avoid overly cautious phrasing such as:

“This suggests that there is a significant likelihood that patients may not respond…”

proposition: “This suggests that many patients may not respond to treatment.”

2/ Is COE-D8 patented? Has it been approved as a therapeutic agent in any country? This should be clearly stated in the introduction or discussion.

3/ Some figures lack information regarding statistical tests and significance indicators. Please standardize this across all figures and legends, specifying p-values, error bars, and the statistical methods used.

4/ (p13) The study involves animal experimentation. A dedicated ethics sub-section should be added to the Methods section with confirmation that all procedures followed current animal welfare regulations and guidelines.

5/ The term “Luria-Bertani” should be corrected to the more accurate name Lysogeny Broth (see: Bertani, J. Bacteriol. 2004).

6/ (P.20) The Data Availability Statement currently indicates “data available on request.” In accordance with PLOS ONE policy, please deposit all genome sequence data and raw data in a public repository (e.g., NCBI SRA, Dryad, Zenodo) and include accession numbers in the manuscript.

7/ (P.21) Were the 93 UPEC isolates derived from 93 independent patients, or were multiple isolates obtained from the same patient at different time points?

8/ You propose MzrA as a resistance-associated target. Were gene expression levels of mzrA or related pathways assessed following COE-D8 exposure?

Reviewer #2: The authors have taken an innovative approach to addressing antibiotic resistance. While the manuscript is generally sound, a few revisions are necessary to improve readability before it can be finalized.

1.In the abstract, there should not be line break. Please change it according to the journal criteria.

2.At the end of the introduction, the author mentioned their findings in a brief. But it would be better to add the objectives of this study which is missing.

Materials and methods:

3. It is mentioned with the reference of COE-D8 synthesized but add in a brief of this protocol easily understandable.

4. The author did not included statistical analysis part which is supposed to be added with reference software the author used.

5. Please ad relevant references in the materials and methods section. References are missing in bioinformatics section

Result:

6. section 3.1 seems combination of your findings and remarks. However, it is supposed to present only the findings from your study and the conclusion marks should be in the discussion part.

7. Figure 1c is cited in the result section and the legend of the figure consists the statistical description and the test name. But it should be included in the description of the result as well and the outcome of test. Please reduce the statements from the 3.1 section and the test findings with proper figure citation.

8. In 3.2 section, the author described reduction of bacterial concentration but the title is related to phylogeny which is missing in the description in this section.

9. Please chnage the whole result section with just your findings. Remarks should be in the discussion part.

10. Please submit your revised manuscript after following the guidelines given by the journal to arrange the information in systematic waym such as reference style, font, arrangment of table, and others.

Reviewer #3: This manuscript presents a study on the antimicrobial potential of a conjugated oligoelectrolyte, COE-D8, and its synergistic combination with fosfomycin against multidrug-resistant uropathogenic Escherichia coli (UPEC). The authors provide a methodologically sound investigation, including in vitro antibacterial efficacy, cytotoxicity evaluation, resistance evolution, mechanistic insights via docking simulations, and in vivo toxicity assessment. The study is timely, addresses a pressing clinical issue, and could contribute significantly to the development of novel antimicrobial strategies.

However, there are some concerns regarding experimental clarity, data interpretation, and presentation that need to be addressed before publication.

1. The docking simulation showed that the binding energy of COE-D8 to the mutant MzrA protein increased by only 0.4 kcal/mol compared to the wild-type. Given the limited precision of docking tools such as AutoDock Vina, this small energy difference may not be sufficient to substantiate MzrA as a key resistance determinant. The authors should clarify whether this minor difference is biologically meaningful and whether additional evidence supports MzrA’s involvement.

2. Apart from MzrA, other genes listed in Table S2 were also found mutated, but their roles in resistance were not evaluated or discussed in sufficient detail. Could the authors speculate or provide functional predictions regarding how these genes might contribute to COE-D8 resistance? For example, a pathway-based discussion would be valuable.

3. The combination of fosfomycin with COE-D8 significantly reduced cytotoxicity in 3T3 cells. How might fosfomycin mitigate the cytotoxicity of COE-D8? This deserves further explanation in the paper. Moreover, the IC50 of 16 μg/mL is still relatively toxic, especially considering the MIC range for UPEC. Have the authors considered chemical modifications of COE-D8 to improve its selectivity and reduce mammalian toxicity?

4. While the authors assessed the in vivo toxicity of COE-D8, the manuscript lacks evaluation of its in vivo antimicrobial efficacy. Demonstrating bacterial burden reduction in infected animals would greatly strengthen the translational value of the study. Please consider including such data or discussing this limitation explicitly.

**********

6. PLOS authors have the option to publish the peer review history of their article (what does this mean?). If published, this will include your full peer review and any attached files.

If you choose “no”, your identity will remain anonymous but your review may still be made public.

**Do you want your identity to be public for this peer review?**  For information about this choice, including consent withdrawal, please see our Privacy Policy.

Reviewer #1: **Yes:** Sebastien Bontemps-Gallo

Reviewer #2: No

Reviewer #3: No

**********

[NOTE: If reviewer comments were submitted as an attachment file, they will be attached to this email and accessible via the submission site. Please log into your account, locate the manuscript record, and check for the action link "View Attachments". If this link does not appear, there are no attachment files.]

While revising your submission, please upload your figure files to the Preflight Analysis and Conversion Engine (PACE) digital diagnostic tool, https://pacev2.apexcovantage.com/. PACE helps ensure that figures meet PLOS requirements. To use PACE, you must first register as a user. Registration is free. Then, login and navigate to the UPLOAD tab, where you will find detailed instructions on how to use the tool. If you encounter any issues or have any questions when using PACE, please email PLOS at figures@plos.org. Please note that Supporting Information files do not need this step.

# Author comment: Author response to Decision Letter 1

**DOI:** 10.1371/journal.pone.0352997.r002

28 Sep 2025

Dear Reviewers,

Thank you very much for your time and for providing valuable and constructive comments on our manuscript entitled ''A Novel COE-D8-Fosfomycin Conjugate Effectively Against First-line Antibiotic Resistant Uropathogenic Escherichia coli ''. We have carefully considered all the suggestions and have revised the manuscript accordingly.

Please find below a summary of our key revisions:

We have addressed all the questions and concerns you raised. Our point-by-point responses are provided in the attached Word document, with all changes highlighted in yellow for your convenience.

All sequencing data have been deposited in a public database, and the relevant accession numbers have been included in the manuscript.

A complete list of all supporting information files has been added to the end of the manuscript.

We have verified that all in-text citations of the supporting information are correct and correspond accurately to the provided files.

We have thoughtfully evaluated the relevant literature you recommended and have cited it where we found it to be directly relevant to our discussion.

The reference list has been thoroughly checked to ensure completeness and strict adherence to the Vancouver style guide.

We have screened the reference list and confirmed that it does not include any retracted articles that are not essential to the study.

As per common practice, the funding information has been moved from the Acknowledgments section to the cover letter.

In short, we are deeply grateful for your valuable guidance, which has greatly strengthened our work and hope that our revisions and responses meet with your approval.

Sincerely,

Ruan Lin

Attachment Submitted filename: Response to Reviewers.docx

# Peer review: Decision Letter 1

**DOI:** 10.1371/journal.pone.0352997.r003

**Authors:** Wondwossen Amogne Degu

8 Feb 2026

PONE-D-25-19542R1

A Novel COE-D8-Fosfomycin Conjugate Effectively Against First-line Antibiotic Resistant Uropathogenic *Escherichia coli*

PLOS One

Dear Dr. Ruan,

Thank you for submitting your manuscript to PLOS ONE. After careful consideration, we feel that it has merit but does not fully meet PLOS ONE’s publication criteria as it currently stands. Therefore, we invite you to submit a revised version of the manuscript that addresses the points raised during the review process.

Please submit your revised manuscript by Mar 25 2026 11:59PM. If you will need more time than this to complete your revisions, please reply to this message or contact the journal office at plosone@plos.org. When you're ready to submit your revision, log on to https://www.editorialmanager.com/pone/ and select the 'Submissions Needing Revision' folder to locate your manuscript file.

Please include the following items when submitting your revised manuscript:

- A letter that responds to each point raised by the academic editor and reviewer(s). You should upload this letter as a separate file labeled 'Response to Reviewers'.
- A marked-up copy of your manuscript that highlights changes made to the original version. You should upload this as a separate file labeled 'Revised Manuscript with Track Changes'.
- An unmarked version of your revised paper without tracked changes. You should upload this as a separate file labeled 'Manuscript'.

-->If you would like to make changes to your financial disclosure, please include your updated statement in your cover letter. Guidelines for resubmitting your figure files are available below the reviewer comments at the end of this letter.

If applicable, we recommend that you deposit your laboratory protocols in protocols.io to enhance the reproducibility of your results. Protocols.io assigns your protocol its own identifier (DOI) so that it can be cited independently in the future. For instructions see: https://journals.plos.org/plosone/s/submission-guidelines#loc-laboratory-protocols. Additionally, PLOS ONE offers an option for publishing peer-reviewed Lab Protocol articles, which describe protocols hosted on protocols.io. Read more information on sharing protocols at https://plos.org/protocols?utm_medium=editorial-email&utm_source=authorletters&utm_campaign=protocols.

We look forward to receiving your revised manuscript.

Kind regards,

Wondwossen Amogne Degu, M.D

Academic Editor

PLOS One

Journal Requirements:

If the reviewer comments include a recommendation to cite specific previously published works, please review and evaluate these publications to determine whether they are relevant and should be cited. There is no requirement to cite these works unless the editor has indicated otherwise.

Please review your reference list to ensure that it is complete and correct. If you have cited papers that have been retracted, please include the rationale for doing so in the manuscript text, or remove these references and replace them with relevant current references. Any changes to the reference list should be mentioned in the rebuttal letter that accompanies your revised manuscript. If you need to cite a retracted article, indicate the article’s retracted status in the References list and also include a citation and full reference for the retraction notice.

[Note: HTML markup is below. Please do not edit.]

Reviewers' comments:

Reviewer's Responses to Questions

**Comments to the Author**

1. If the authors have adequately addressed your comments raised in a previous round of review and you feel that this manuscript is now acceptable for publication, you may indicate that here to bypass the “Comments to the Author” section, enter your conflict of interest statement in the “Confidential to Editor” section, and submit your "Accept" recommendation.

Reviewer #1: All comments have been addressed

Reviewer #2: All comments have been addressed

Reviewer #3: All comments have been addressed

**********

2. Is the manuscript technically sound, and do the data support the conclusions?

The manuscript must describe a technically sound piece of scientific research with data that supports the conclusions. Experiments must have been conducted rigorously, with appropriate controls, replication, and sample sizes. The conclusions must be drawn appropriately based on the data presented.

Reviewer #1: Yes

Reviewer #2: Yes

Reviewer #3: Yes

**********

3. Has the statistical analysis been performed appropriately and rigorously?

Reviewer #1: Yes

Reviewer #2: N/A

Reviewer #3: Yes

**********

4. Have the authors made all data underlying the findings in their manuscript fully available?

The PLOS Data policy requires authors to make all data underlying the findings described in their manuscript fully available without restriction, with rare exception (please refer to the Data Availability Statement in the manuscript PDF file). The data should be provided as part of the manuscript or its supporting information, or deposited to a public repository. For example, in addition to summary statistics, the data points behind means, medians and variance measures should be available. If there are restrictions on publicly sharing data—e.g. participant privacy or use of data from a third party—those must be specified.

Reviewer #1: Yes

Reviewer #2: Yes

Reviewer #3: Yes

**********

5. Is the manuscript presented in an intelligible fashion and written in standard English?

PLOS ONE does not copyedit accepted manuscripts, so the language in submitted articles must be clear, correct, and unambiguous. Any typographical or grammatical errors should be corrected at revision, so please note any specific errors here.

Reviewer #1: Yes

Reviewer #2: Yes

Reviewer #3: Yes

**********

6. Review Comments to the Author

Please use the space provided to explain your answers to the questions above. You may also include additional comments for the author, including concerns about dual publication, research ethics, or publication ethics. (Please upload your review as an attachment if it exceeds 20,000 characters)

Reviewer #1: On page 61: mzrA should be italicized in the sentence, “It should be acknowledged that a limitation of this study is the lack of gene expression analysis of mzrA.”

Reviewer #2: The authors have adequately addressed all of my comments and concerns. The revisions have improved the clarity and scientific rigor of the manuscript. I find the responses satisfactory, and the manuscript entitled “A Novel COE-D8-Fosfomycin Conjugate Effectively Against First-Line Antibiotic-Resistant Uropathogenic Escherichia coli” is now acceptable in its current form

Reviewer #3: (No Response)

**********

7. PLOS authors have the option to publish the peer review history of their article (what does this mean?). If published, this will include your full peer review and any attached files.

If you choose “no”, your identity will remain anonymous but your review may still be made public.

**Do you want your identity to be public for this peer review?**  For information about this choice, including consent withdrawal, please see our Privacy Policy.

Reviewer #1: **Yes:** Sébastien Bontemps-Gallo

Reviewer #2: No

Reviewer #3: No

**********

[NOTE: If reviewer comments were submitted as an attachment file, they will be attached to this email and accessible via the submission site. Please log into your account, locate the manuscript record, and check for the action link "View Attachments". If this link does not appear, there are no attachment files.]

To ensure your figures meet our technical requirements, please review our figure guidelines: https://journals.plos.org/plosone/s/figures

You may also use PLOS’s free figure tool, NAAS, to help you prepare publication quality figures: https://journals.plos.org/plosone/s/figures#loc-tools-for-figure-preparation.

NAAS will assess whether your figures meet our technical requirements by comparing each figure against our figure specifications.

# Author comment: Author response to Decision Letter 2

**DOI:** 10.1371/journal.pone.0352997.r004

30 Mar 2026

Reviewer #1: On page 61: mzrA should be italicized in the sentence, “It should be acknowledged that a limitation of this study is the lack of gene expression analysis of mzrA.”

Author Response: We sincerely appreciate your professional and detailed comment on our manuscript. We have fully revised the manuscript in strict accordance with your suggestion: the gene name mzrA in the corresponding sentence on page 61 has been formatted in italics, which fully complies with the gene nomenclature guidelines of PLOS ONE.Meanwhile, we have highlighted this modification in yellow in the Revised Manuscript with Track Changes for your convenient review and verification. In addition, taking this format requirement as the standard, we have conducted a comprehensive cross-check of the writing format of all gene names and species scientific names throughout the manuscript, to ensure the uniform format of the full text and full compliance with the journal's submission formatting specifications.

Reviewer #2: The authors have adequately addressed all of my comments and concerns. The revisions have improved the clarity and scientific rigor of the manuscript. I find the responses satisfactory, and the manuscript entitled “A Novel COE-D8-Fosfomycin Conjugate Effectively Against First-Line Antibiotic-Resistant Uropathogenic Escherichia coli” is now acceptable in its current form

Author Response: We sincerely thank you for your recognition of our research work, as well as all the professional comments and valuable suggestions you have put forward during the review process. Your suggestions have greatly helped us improve the content of the manuscript, and enhance the clarity of presentation and scientific rigor of this study.We have carefully reviewed all of your review comments, and re-checked the research content, data presentation and narrative expression of the manuscript in an all-round way, to ensure the rigor, standardization and integrity of the manuscript content. We once again sincerely appreciate your valuable time and effort dedicated to the review of our manuscript.

Reviewer #3: (No Response)

Author Response: We have carefully checked all the review feedback from Reviewer #3, and there are no comments or suggestions requiring revision. We sincerely appreciate the time and effort that Reviewer #3 has dedicated to the review of our manuscript.

Attachment Submitted filename: 20260325_submit_Response to Reviewers.docx

# Peer review: Decision Letter 2

**DOI:** 10.1371/journal.pone.0352997.r005

**Authors:** Vinothkannan Ravichandran

14 Jun 2026

A Novel COE-D8-Fosfomycin Conjugate Effectively Combats First-line Antibiotic Resistant Uropathogenic *Escherichia coli*

PONE-D-25-19542R2

Dear Dr. Ruan,

We’re pleased to inform you that your manuscript has been judged scientifically suitable for publication and will be formally accepted for publication once it meets all outstanding technical requirements.

Within one week, you’ll receive an e-mail detailing the required amendments. When these have been addressed, you’ll receive a formal acceptance letter and your manuscript will be scheduled for publication.

An invoice will be generated when your article is formally accepted. Please note, if your institution has a publishing partnership with PLOS and your article meets the relevant criteria, all or part of your publication costs will be covered. Please make sure your user information is up-to-date by logging into Editorial Manager at Editorial Manager® and clicking the ‘Update My Information' link at the top of the page. For questions related to billing, please contact billing support.

If your institution or institutions have a press office, please notify them about your upcoming paper to help maximize its impact. If they’ll be preparing press materials, please inform our press team as soon as possible -- no later than 48 hours after receiving the formal acceptance. Your manuscript will remain under strict press embargo until 2 pm Eastern Time on the date of publication. For more information, please contact onepress@plos.org.

Kind regards,

Vinothkannan Ravichandran

Academic Editor

PLOS One

Additional Editor Comments (optional):

Dear Authors,

The revised manuscript has been carefully evaluated. The authors have made substantial and satisfactory revisions that adequately address the major concerns raised during the previous review.

In its current form, the manuscript demonstrates improved clarity, coherence, and scientific rigor, and is now suitable for publication.

Reviewers' comments:

Reviewer's Responses to Questions

**Comments to the Author**

1. If the authors have adequately addressed your comments raised in a previous round of review and you feel that this manuscript is now acceptable for publication, you may indicate that here to bypass the “Comments to the Author” section, enter your conflict of interest statement in the “Confidential to Editor” section, and submit your "Accept" recommendation.

Reviewer #1: (No Response)

Reviewer #2: All comments have been addressed

Reviewer #3: All comments have been addressed

**********

2. Is the manuscript technically sound, and do the data support the conclusions?

The manuscript must describe a technically sound piece of scientific research with data that supports the conclusions. Experiments must have been conducted rigorously, with appropriate controls, replication, and sample sizes. The conclusions must be drawn appropriately based on the data presented.

Reviewer #1: (No Response)

Reviewer #2: Yes

Reviewer #3: Yes

**********

3. Has the statistical analysis been performed appropriately and rigorously?

Reviewer #1: (No Response)

Reviewer #2: Yes

Reviewer #3: Yes

**********

4. Have the authors made all data underlying the findings in their manuscript fully available?

The PLOS Data policy requires authors to make all data underlying the findings described in their manuscript fully available without restriction, with rare exception (please refer to the Data Availability Statement in the manuscript PDF file). The data should be provided as part of the manuscript or its supporting information, or deposited to a public repository. For example, in addition to summary statistics, the data points behind means, medians and variance measures should be available. If there are restrictions on publicly sharing data—e.g. participant privacy or use of data from a third party—those must be specified.

Reviewer #1: (No Response)

Reviewer #2: Yes

Reviewer #3: Yes

**********

5. Is the manuscript presented in an intelligible fashion and written in standard English?

PLOS ONE does not copyedit accepted manuscripts, so the language in submitted articles must be clear, correct, and unambiguous. Any typographical or grammatical errors should be corrected at revision, so please note any specific errors here.

Reviewer #1: (No Response)

Reviewer #2: Yes

Reviewer #3: Yes

**********

6. Review Comments to the Author

Please use the space provided to explain your answers to the questions above. You may also include additional comments for the author, including concerns about dual publication, research ethics, or publication ethics. (Please upload your review as an attachment if it exceeds 20,000 characters)

Reviewer #1: Although all three reviewers had approved the manuscript, the R2 version contains substantial modifications and rewriting. Would it be possible to revert to the version approved in R1, with the only modification being the gene mzrA in italics?

Reviewer #2: Thank you to all the others. The current format of this paper is now acceptable and scientific. The author has addressed all the comments adequately.

Reviewer #3: (No Response)

**********

7. PLOS authors have the option to publish the peer review history of their article (what does this mean?). If published, this will include your full peer review and any attached files.

If you choose “no”, your identity will remain anonymous but your review may still be made public.

**Do you want your identity to be public for this peer review?**  For information about this choice, including consent withdrawal, please see our Privacy Policy.

Reviewer #1: No

Reviewer #2: No

Reviewer #3: No

**********

# Editor report: Acceptance letter

**DOI:** 10.1371/journal.pone.0352997.r006

**Authors:** Vinothkannan Ravichandran

PONE-D-25-19542R2

PLOS One

Dear Dr. Guo,

I'm pleased to inform you that your manuscript has been deemed suitable for publication in PLOS One. Congratulations! Your manuscript is now being handed over to our production team.

At this stage, our production department will prepare your paper for publication. This includes ensuring the following:

* All references, tables, and figures are properly cited

* All relevant supporting information is included in the manuscript submission,

* There are no issues that prevent the paper from being properly typeset

You will receive further instructions from the production team, including instructions on how to review your proof when it is ready. Please keep in mind that we are working through a large volume of accepted articles, so please give us a few days to review your paper and let you know the next and final steps.

Lastly, if your institution or institutions have a press office, please let them know about your upcoming paper now to help maximize its impact. If they'll be preparing press materials, please inform our press team within the next 48 hours. Your manuscript will remain under strict press embargo until 2 pm Eastern Time on the date of publication. For more information, please contact onepress@plos.org.

You will receive an invoice from PLOS for your publication fee after your manuscript has reached the completed accept phase. If you receive an email requesting payment before acceptance or for any other service, this may be a phishing scheme. Learn how to identify phishing emails and protect your accounts at https://explore.plos.org/phishing.

If we can help with anything else, please email us at customercare@plos.org.

Thank you for submitting your work to PLOS ONE and supporting open access.

Kind regards,

PLOS ONE Editorial Office Staff

on behalf of

Dr. Vinothkannan Ravichandran

Academic Editor

PLOS One
